# Ecological opportunity and predator–prey interactions: linking eco-evolutionary processes and diversification in adaptive radiations

**DOI:** 10.1098/rspb.2017.2550

**Published:** 2018-03-07

**Authors:** Mikael Pontarp, Owen L. Petchey

**Affiliations:** 1 Department of Evolutionary Biology and Environmental Studies, University of Zurich, Winterthurerstrasse 190, 8057 Zurich, Switzerland; 2 Department of Ecology and Environmental Science, Umeå University, 90187 Umeå, Sweden

**Keywords:** adaptive radiation, macroevolution, community patterns, competition, predation, ecological speciation

## Abstract

Much of life's diversity has arisen through ecological opportunity and adaptive radiations, but the mechanistic underpinning of such diversification is not fully understood. Competition and predation can affect adaptive radiations, but contrasting theoretical and empirical results show that they can both promote and interrupt diversification. A mechanistic understanding of the link between microevolutionary processes and macroevolutionary patterns is thus needed, especially in trophic communities. Here, we use a trait-based eco-evolutionary model to investigate the mechanisms linking competition, predation and adaptive radiations. By combining available micro-evolutionary theory and simulations of adaptive radiations we show that intraspecific competition is crucial for diversification as it induces disruptive selection, in particular in early phases of radiation. The diversification rate is however decreased in later phases owing to interspecific competition as niche availability, and population sizes are decreased. We provide new insight into how predation tends to have a negative effect on prey diversification through decreased population sizes, decreased disruptive selection and through the exclusion of prey from parts of niche space. The seemingly disparate effects of competition and predation on adaptive radiations, listed in the literature, may thus be acting and interacting in the same adaptive radiation at different relative strength as the radiation progresses.

## 1. Introduction

It is well known that ecological and evolutionary time scales can overlap and that community richness can be a consequence of both ecological and evolutionary processes acting in concert [1]. These ideas are supported by empirical studies showing that diversity of various organisms has arisen through adaptive radiations [2–4]. Such diversification is thought to be facilitated by ecological opportunity and niche availability through colonization of a novel environment or mutations that lead to innovations [2,5].

Several mechanisms have been suggested for the link between ecological opportunity and adaptive radiations [2]. Theory for ecological speciation shows that frequency-dependent competition for common resources can drive diversification [6–8]. Interspecific competition can thus be one of the main drivers of adaptive radiations [9–11]. Conversely, competition for niche space also affects niche availability, which is one of the major prerequisites for adaptive radiations [12–14]. Competition can also decrease population size, which in turn may lead to reduced genetic variation, fewer beneficial mutations, reduced disruptive selection and ultimately low diversity [4,15,16]. Such effects may underlie empirical results showing that competition can both promote [17] and reduce [18] diversification, but to fully understand such contrasting results, a better link between radiations and competition is required.

Predation can also drive adaptive radiations [5]. Divergence can occur when prey adapt in different ways to predation by a common predator [19,20]. Theory also supports the idea that trophic interactions can induce disruptive selection on prey populations and thus drive evolutionary branching [21–23]. Conversely, predation may reduce prey population size which can reduce prey diversification owing to reasons explained above. The empirical support for one or the other of such effects of predation is, however, limited as few studies have focused on this issue [5,24,25].

**Figure 1.**
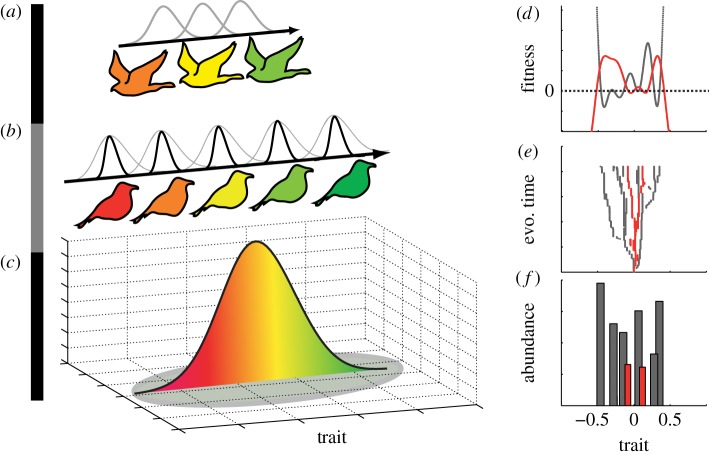
Model illustration (*a*–*c*) and example of model output (*d*–*f*). A species pool of top consumers (*a*) with some trait *z* (e.g. birds of prey with body size *z*) and a pool of competitive consumers (*b*) with trait *u* (e.g. granivorous birds with beak size *u*) that interact on an island (*c*) defined by some implicit resource distribution with peak abundance as *u*_opt_ and width *σ_K_*. The three trophic levels are distributed on the same trait dimension (e.g. size) here illustrated by colour. Competition between species is dictated by their niche width (black and grey Gaussian kernels), and we assume that populations with similar traits interact more than less similar ones. The invasion fitness of a mutant is thus a function of its trait-matching to its resources, the traits of its competitors on the same trophic level and their niche widths. We simulate adaptive radiations (*e*) and community data (*f*) with the assumption of ecological opportunity by seeding the system with monomorphic populations with trait value equal to *u*_opt_. From this starting point at each evolutionary time step we computed community equilibrium, we allowed for mutations, computed mutant invasion fitness (*d*), and we either added the mutant population to the community or replaced the mutating population with the mutant population. Grey and red colour in (*d*–*f*) denote data associated with prey and predators, respectively.

Each of the effects presented above makes sense when viewed in isolation. However, their combined effect is largely unknown, which makes the full mechanistic link between ecological opportunity and adaptive radiations elusive [2]. Theory that links ecological and evolutionary processes on the micro-scale with macroevolutionary patterns (see examples of such patterns in [3,4,17]) is thus needed [26]. Along these lines, evolutionary radiations in predator–prey systems have been investigated [22,23,27,28] but much is still unknown about the mechanisms behind adaptive radiations in trophic communities. With this in mind, we aim to reconcile theory on ecological speciation and some of the seemingly disparate causalities between ecological interactions and adaptive radiations. We adopt a trait-based [7,29,30] adaptive dynamics approach [6] and we construct a simulation model based on the assumption that ecological opportunity for diversification exists (figure 1). As a baseline for our investigations, we first study how the degree of competition between competitor species (defined through their niche width) affects diversification of a community of only competitors. Then, as we are interested in quantifying the effect of predation on the diversification of competitors, we investigate how predator properties like niche width, attack rate and predator mutation rate affect predator–prey co-evolution in adaptive radiations. We use this approach to test two *a priori* predictions derived from current theoretical and empirical work. First, if species niche width of the radiating organism is narrow in relation to the total niche availability then adaptive radiations will be facilitated [6,7,22,31] and the radiation will continue with declining diversification rate as niche space is filling up and population sizes decline [32–35]. This scenario has been studied before, with similar models. We used it as a baseline for our extended analysis including both competition and trophic interactions. Second, predator niche width, attack efficiency and mutation probability may affect prey radiations negatively through decreased divergent selection on prey and reduced prey population sizes [4,36]. To test these predictions, we follow the radiation process throughout evolutionary time and quantify community metrics like species richness, trait distributions, population size, competition strength and predation.

## 2. Ecological model

We use the generalized Lotka–Volterra (GLV) model as the basis for the eco-evolutionary dynamics of prey and predator populations [37] (figure 1). The ecological dynamics, in *per capita* form, of *n* prey populations and *p* predator populations are described as:2.1
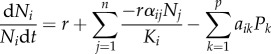
and2.2
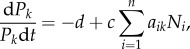
for *i* = 1 to *n*, *k* = 1 to *p* and where *N_i_* and *P_k_* denote prey and predator population size respectively. The parameter *r* is the intrinsic growth rate, and *K_i_* denotes the carrying capacity of prey population *i*. The parameter *α_ij_* denotes competition between prey populations' *i* and *j*. Parameter *d* is the intrinsic death rate of the predators, *c* is the conversion coefficient from prey to predator and *a_ik_* denotes the rate of attack from predator *k* on prey *i*.

The model in its basic form, as it is formulated above, does not include trait dependent interactions or explicit resource utilization. However, similar to other trait-based models [7,29,30] we expand on this model and describe the competitive community with dynamic vectors **N** and **P**, representing prey and predator population abundances respectively. We also introduce static (on ecological time scale) vectors **u** and **z**, representing the prey and predator population traits. We then reformulate carrying capacity (*K_i_*), the prey interactions (*α_ij_*) and predator–prey interactions (*a_ik_*) as trait dependent functions:2.3
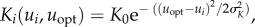
2.4
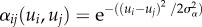
2.5
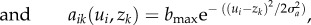
where *K_i_*(*u_i_*, *u*_opt_) represents the carrying capacity for a monomorphic population of prey individuals with trait value *u_i_* in a habitat characterized by a resource distribution with its peak resource availability at the point *u*_opt_. For simplicity, but without loss of generality, we set *u*_opt_ = 0 throughout our analysis. *K*_0_ denotes the maximal carrying capacity (at *u* = *u*_opt_) and it follows from equation (2.3) that the resource availability declines symmetrically as *u* deviates from *u*_opt_ according to the width of the resource distribution (*σ_K_*).

Equation (2.4) models the interaction coefficient, *α_ij_*(*u_i_*, *u_j_*), between the focal prey population (defined by its trait *u_i_*) and its competitors (defined by their traits *u_j_*). Here, we standardize the competition coefficients so that, for a focal population *i*, *α_ii_* = 1 and 0 < *α_ij_* < 1 (*u_i_* ≠ *u_j_*). *σ_α_* determines the degree of competition between individuals given certain utilization traits and can thus be viewed as the niche width of the prey.

Equation (2.5) models the interaction, *a_ik_*(*u_i_*, *z_k_*), between a focal predator population *k* with trait value *z* and a prey population *i* with trait value *u*. The parameter *b*_max_ denotes the maximum attack rate obtained when *u_i_* = *z_k_* and this rate then falls of symmetrically as *u_i_* deviates from *z_k_* according to a Gaussian function with variance *σ_a_*. Similar to the *σ_α_* parameter, *σ_a_* can be viewed as the niche width of the predator. From the above, it follows that our full trait-based ecological model is formulated as:2.6
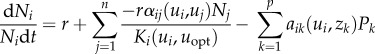
and2.7
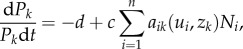
and it also follows that prey populations compete for resources and predators consume prey in a spatially distinct and homogeneous habitat and local resources are distributed in the same trait dimension as the predator and prey resource utilization trait. Similar to, for example, Doebeli & Dieckmann [36] we assume that consumer–resource and consumer–consumer trait matching dictates resource utilization and competition respectively. Given these general assumptions and similar to previous community models [9,10,38], the *per capita* growth (fitness) of a focal competitor individual associated with a given population is thus a function of its resource utilization trait, the abundance of the individual's own population, the local resource distribution and the abundance of all other populations competing for the same resources. The fitness of a predator is a function of its trait, the traits and abundance of its prey and the traits and abundance of other predators to which the focal predator competes.

## 3. Evolutionary analysis

The fitness landscape in trait space for both predator and prey will be determined by the distribution of species and their abundances as well as the resource distribution in trait space. The resource utilization traits (*u* and *z*) are under selection with the potential to evolve as a response to the ecological properties of the system. Fitness will be low in parts of trait space where many abundant populations occur owing to competition, even though the underlying resources may be abundant initially. Contrary, fitness can be positive in parts of trait space where resources may be scarce if there is little or no competition for those resources. We adopt the adaptive dynamics framework [6,39] to formulate trait dependent fitness for an arbitrary predator and prey mutant mathematically as:3.1
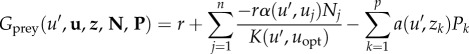
and3.2
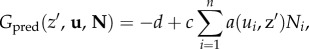
where *u*′ denotes the trait value of the mutant prey and *z*′ denotes the trait value of a mutant predator. The vectors **u**, **z**, **N** and **P** are defined as above containing the resident community trait distributions and abundances.

The expressions stated in equations (3.1) and (3.2) are general, describing the fitness of any focal species conditioned on its traits and the traits of other species with whom it may interact. As our study is focused on adaptive radiation under the assumption of ecological opportunity, we start our evolutionary analysis with only one (in the prey only case) or two (predator–prey case). Focusing on our baseline prey analysis for now and following adaptive dynamics theory, the slope of the prey fitness functions presented above dictates the speed of prey evolution. For our model the slope, or the prey fitness gradients, is formulated as:3.3
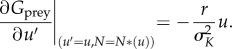
This partial derivative describes eco-evolutionary dynamics when it is introduced in the generally formulated canonical equation first presented by Dieckmann & Law [40]:3.4
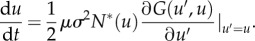
Equation (3.4) describes how the value of an ecological trait (*u*) evolves depending on the *per capita* mutation probability (*µ*, related to our parameters *µ*_prey_ and *µ*_pred_), the variance of mutation size (*σ*, related to our *σ*_mut_), the population size at equilibrium (N*) and the selection gradient (equation (3.3)). It follows that the fitness gradient is positive for *u* < 0, negative for *u* > 0 and zero at *u* = *u*_opt_ = 0.

Differentiating equation (3.3) with respect to *u* gives us:3.5
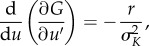
which tells us that *u* = 0 is always a convergent stable evolutionary singular point. A population of individuals with trait *u* away from zero will always evolve towards *u* = 0. What happens when the population has reached *u* = 0 is model dependent and can be analysed through the second order partial derivative of the prey fitness function, with respect to *u′*:3.6
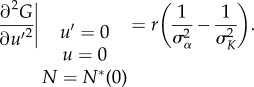
Equation (3.6) tells us that selection is disruptive and evolutionary branching can occur if mutant populations are allowed to invade and if *σ_α_* < *σ_K_*. If *σ_α_* > *σ_K_* the selection is stabilizing and no branching will occur [36].

Now, introducing the predators with trait value *z* = *u*_opt_ we analyse the first evolutionary singular point of the predator–prey system by recalculating the derivatives presented in equations (3.3), (3.5) and (3.6) with the predator included. First, we need to find the expressions for the prey and the predator populations at *u* = *z* = *u*_opt_. Prey equilibrium (N*) is easily computed by solving equation (2.7) with respect to *N*. By substituting *N* for N* in equation (2.6) and solving with respect to *P* we get P*. Equilibrium population sizes for our model then becomes:3.7
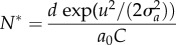
and3.8
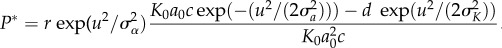


The expressions in equations (3.7) and (3.8) can now be substituted into equation (3.1) and the second-order partial derivatives computed above now becomes:3.9
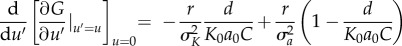
and3.10
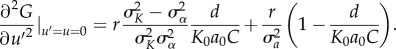


Equations (3.9) and (3.10) tell us that the convergent stable evolutionary branching point in the prey system at *u*_opt_ becomes an unstable repellor for large parts of parameter space, when a predator with trait value *z* = *u*_opt_ is introduced. This result is intuitive, prey tends to evolve away, in trait space, from predators unless the resource peak is very large (e.g. high *K*_0_) or if the predator is weak (e.g. high *d* or low *a*_0_ and *c*).

## 4. Simulation algorithm and parameter values

The analytical investigation presented above gives us an idea of how the system behaves initially, but it tells us little about adaptive radiations. We use simulations to study adaptive radiations in competitive prey communities without predators (our reference community) and we compare these reference communities to co-radiating predator–prey communities. We start by setting up the model described above, we implement the assumption of ecological opportunity by seeding the system with one (in scenarios without predators) or two (in the predator–prey scenario) monomorphic population(s) with trait value equal to *u*_opt_ and we compute the equilibrium population size by solving our ecological model numerically (equations (2.6) and (2.7)). We refer to the population(s) with positive abundance in the system at ecological equilibrium as resident population(s) from now on. From this starting point, with resident population(s) at equilibrium, we then ask whether a mutant with trait value *u*′ or *z*′ can invade. Similar to, for example, Ito & Dieckmann [31] for each evolutionary time step we compute community equilibrium, we allow for mutations, compute mutant invasion fitness, mutant and resident mutual invasibility and we either add the mutant population to the community or replace the mutating population with the mutant population.

We compute the equilibrium population sizes by integrating over equations (2.6) and (2.7) until equilibrium or a steady state has been reached. Then, we introduce mutants. Populations mutate according to the product of the population size and mutation probability (*μ*_prey_ and *μ*_pred_). Abundant populations are thus more likely to mutate than less abundant ones. More specifically, a single mutant is drawn at each evolutionary time step with probability weighted by population sizes (related to the total number of individuals in the system) and the mutation probabilities. We modelled mutation size for both predators and prey as a random trait value drawn from a normal distribution with mean equal to the trait value of the mutating population and a variance (*σ*_mut_) equal to 0.02. Mutation probability for the prey (*μ*_prey_) was kept constant at 0.01. We compute invasions fitness by solving equation (3.1) for a prey mutant and equation (3.2) for a predator mutant. If the mutant has positive invasion fitness, we continue our analysis with a mutual invasibility test. This means that the mutant is allowed to replace the resident population, equilibrium is recalculated and the invasion fitness of a population with the resident morph is quantified, using the same procedure as described above. If the mutant invasion fitness is positive but mutual invasibility does not exist, the mutant will replace the resident. However, if mutual invasibility does exist, the resident and the mutant can coexist. After the mutant is either introduced alongside the resident or replaced the resident, we recalculate the equilibrium, removing populations that may have gone extinct owing to the introduction of the new population. We thus progress into the next evolutionary step, repeating the whole procedure and we run our simulations for 3000 evolutionary steps. As a robustness check, additional simulations were also run with 5000 evolutionary steps (electronic supplementary material, appendix 1).

For each evolutionary step, we also assigned each population to a species ID using a trait-based speciation definition (see also [9,10]). We define species as populations having common descent and a continuous distribution of traits (no gaps in the trait distribution > 3* *σ*_µ_). When a gap > 3* *σ*_µ_ was detected in the trait distribution within an existing species, it was considered a speciation event (i.e. one species branching into two). Although somewhat arbitrary, this limit of 3* *σ*_µ_ makes biological sense as it is large enough to prevent speciation by only a few mutations. By registering the time and origin of all speciation events as well as trait distributions and abundance throughout evolutionary history we have all the information required to follow the dynamics of diversity, phylogenetic and phenotypic community structure.

In our endeavour to understand radiations in our reference competitive community, we simulate radiations with different prey niche widths (*σ_α_* = 0.1–0.7). Prey niche width needs be considered in relation to the width of the resource distribution (*σ_K_*). If *σ_α_* is larger than or close to the width of the resource distribution *σ*_K_ (here set as a constant = 1), competition strength will be high even among populations using opposite ends of the local resource spectrum. Consequently, there will only be room (regarding niche space) for one population and no branching will occur in the local community [9,10]. If, on the other hand, the biotic niche width is narrower (*σ_α_* < *σ_K_*), then local evolutionary branching is facilitated, driving prey speciation [6,7]. When we study predatory effects on competitive prey radiations we investigate the effect of predator niche widths (*σ_a_* = 0.1–0.7) which is a parameter that should be interpreted in the same way as *σ_α_* with the exception that predators consume discrete resources (prey populations) rather than a continuous resource distribution defined through *K*_0_, *u*_opt_ and *σ_K_*. We also analysed a range of predator efficiency (*b*_max_ = 0.0001–0.0007) and we varied predator mutation probability (*μ*_pred_ = 0.005–0.1). Constant model parameters for the simulations were: *K*_0_ = 10 000; *σ_K_* = 1; *r* = 1; *d* = 0.2; *c* = 0.3. All constants, as well as the ranges in the analysed parameter space, were chosen to produce diverse enough communities to analyse adaptive radiations within reasonable computational time. Parameters *r* and *d* were chosen to get stable ecological dynamics at simulation initiation. With this being said, we do run simulations with different *r* and *d* as a robustness check (electronic supplementary material, appendix 1).

## 5. Results

**Figure 2.**
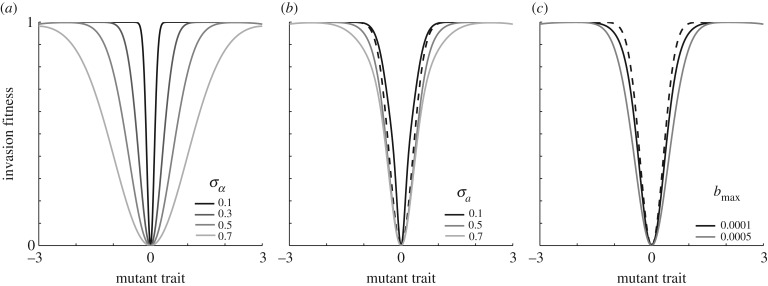
Prey fitness landscape at ecological equilibrium after simulation initiation and before first branching. (*a*) Prey fitness landscape for a prey system with prey niche width ranging from 0.1 to 0.7. (*b*) Prey fitness landscape for a predator–prey system with prey niche width equal to 0.3 and predator niche width ranging from 0.1 to 0.7. (*c*) Prey landscape for a predator–prey system with predator efficiency ranging from 0.0001 to 0.0005. Dashed lines in (*b*) and (*c*) illustrate predator landscape without a predator. If nothing else is stated parameters and traits were set to: *u* = 0; *z* = 0; *u*_opt_ = 0; *K*_0_ = 10 000; *σ_K_* = 1; *r* = 1; *σ_α_* = 0.3; *d* = 0.2; *c* = 0.3; *σ_a_* = 0.5; *b*_max_ = 0.0001.

As expected, we see a clear relationship between prey niche width and the possibility for branching (figure 2). The population finds itself at a fitness minimum, a branching point, and given that the prey niche width is smaller than the width of the resource distribution [36], frequency-dependent competition for resources makes all mutants beneficial. As formulated by the canonical equation (equation (3.4)) the speed of which that evolutionary change occurs is dependent on the fitness gradient, population sizes, and mutation probability. We also find that the curvature of the fitness landscape at the branching point depends on prey niche width, suggesting that prey branching can be rapid, such that incipient species diverge fast, when niche width is narrow (figure 2*a*).

In the predator–prey system, also initiated with traits at resource optimum, we find a similar prey branching point, and the curvature at the fitness minimum is affected by predator niche width and efficiency (figure 2*b*,*c* and equation (3.10)). For example, our numerical analysis shows that when predator niche width is narrower, the curvature at the branching point is steeper compared to when predators were absent. Again, the speed at which that evolutionary change occurs is dependent on the fitness gradient, population sizes, and mutation probability which suggests that predators can promote and speed up prey branching. When predator niche width is wide, the prey fitness landscape is, however, shallower, suggesting that predators can slow down prey branching. Predator efficiency also affects the disruptive selection acting on prey by making the fitness landscape shallower, and a high efficiency interrupts speciation more than a less efficient predator (figure 2*c*).

**Figure 3.**
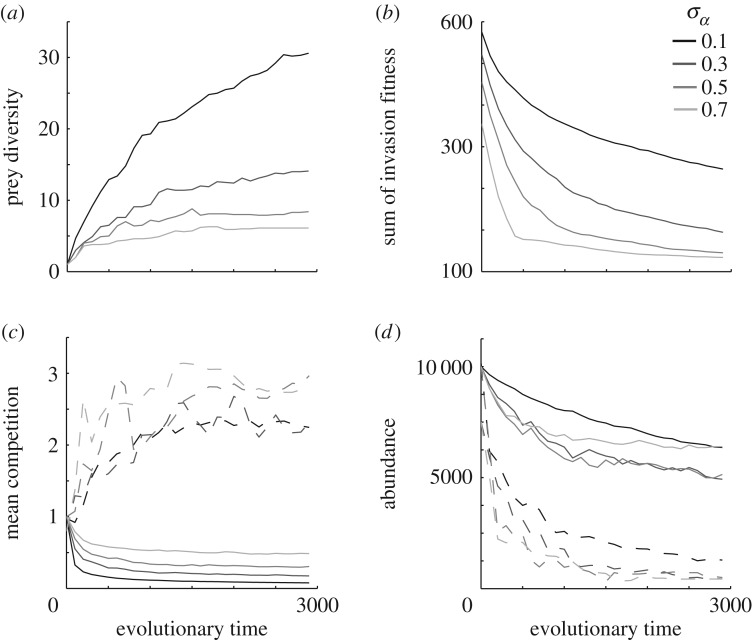
Prey community adaptive radiation data. (*a*) Prey diversity (mean over 10 replicated simulations) increase with evolutionary time and prey diversification rate, as well as prey diversity, is negatively related to prey niche width. (*b*) Niche availability, measured as the sum of invasion fitness for prey mutants evenly distributed between −3 and 3 in trait space instead decrease with evolutionary time and increased niche width. (*c*,*d*) Mean competition in the whole community (*c*, solid lines) and mean carrying capacity (*d*, solid lines) decrease as diversification progress and species evolve into the peripheral parts of trait space but the mean realized competition for a given species (*c*, dashed lines) increase owing to niche packing. Realized competition and low carrying capacity in peripheral parts of trait space combine to decrease population size (*d*, dashed lines) which in turn can decrease the evolutionary rate. If nothing else is stated parameters and traits were set to: *u*_opt_ = 0; *K*_0_ = 10 000; *σ_K_* = 1; *r* = 1; *σ_α_* = 0.1; *μ*_prey_ = 0.01.

The results presented above can to some extent be derived from the adaptive dynamics theory literature (e.g. [6,7,21,22]). However, adaptive dynamics theory focuses largely on the details of evolutionary singular points and while they give an idea of how ecological interactions affects diversification, they do not provide full understanding or predictions about adaptive radiations, especially co-radiating predator–prey radiations (but see [22]). Our results on prey adaptive radiations show that diversity builds up over evolutionary time with a negative relationship between diversification rate and prey niche width (figure 3*a*). Radiation occurs, and the community spreads out in trait space and niche availability, measured as the sum of positive invasion fitness for prey mutants evenly distributed between −3 and 3 in trait space, decrease (figure 3*b*). A consequence of the radiation spreading out in trait space is that mean competition, computed as the mean of all elements in the community matrix, decreases (figure 3*c*). The decrease in available niche space leads to increased niche packing which results in increased competition experiences by the species, measured as the mean of the row sums of the community matrix (figure 3*c*). Mean carrying capacity and mean population size also decrease as diversification progresses (figure 3*d*). We thus find two processes that combine to decrease diversification rate: (i) niche availability is decreased such that the fitness landscape becomes shallower, and (ii) population size decreases and thus reduces evolvability (related to N* in equation (3.4)). Both processes are also directly influenced by the niche width of the radiating organism.

**Figure 4.**
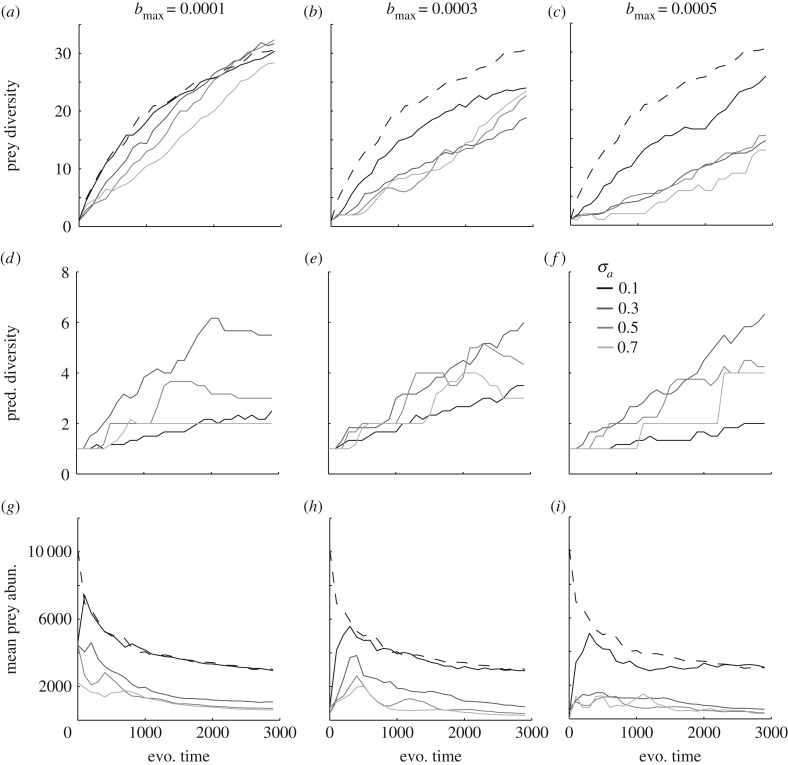
Predator–prey community adaptive radiation data. Prey diversity (mean over 5 replicated simulations) and diversification rate are in general lower in predator–prey radiations (solid lines) compared to prey radiations (dashed line) (*a*–*c*). Prey diversity and diversification rate are also negatively related to predator niche width and efficiency (*a*–*c*). Note the increase in predator efficiency with panel columns. Predators also radiate, and predator diversity is positively related to prey diversity (*d*–*f*). Mean prey abundance decreases with evolutionary time, and prey abundance tends to be higher in prey radiations (dashed line) than in predator–prey radiations (solid lines) (*g*–*i*). If nothing else is stated parameters and traits were set to: *u*_opt_ = 0; *K*_0_ = 10 000; *σ_K_* = 1; *r* = 1; *σ_α_* = 0.1; *d* = 0.2; *c* = 0.3; *μ*_pred_ = 0.01; *μ*_prey_ = 0.01.

**Figure 5.**
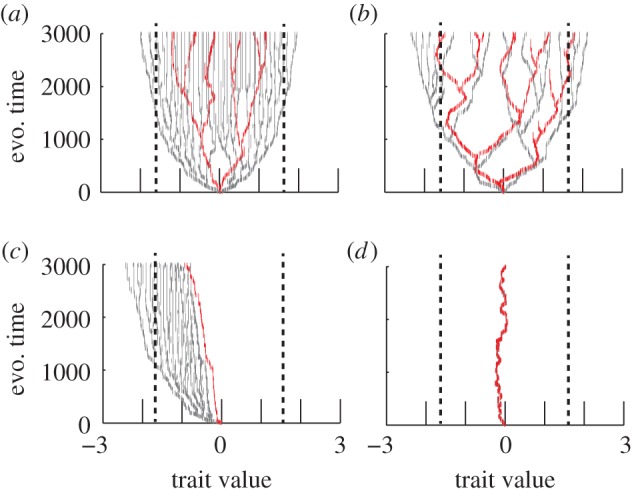
Predator–prey adaptive radiations across parameter space. Co-radiation at intermediate predator niche width (*σ_a_* = 0.3), low predator mutation probability (*μ*_pred_ = 0.005) and low (*a*) and high (*b*) predator efficiency (*b*_max_ = 0.0001 (*a*) and 0.0005 (*b*)). (*c*) Predators excluding the prey from parts of trait space when the predator's efficiency is large (*b*_max_ = 0.0007), niche width is low (e.g. *σ_a_* < 0.1) and mutation probability is low (*μ*_pred_ = 0.005). (*d*) High values of predator mutation probability (*μ*_pred_ = 0.1), in combination with high predator efficiency (*b*_max_ = 0.0007) interrupts the branching all together, only one predator and one prey population co-evolve in trait space with the predator trait (red) completely overlapping the prey (grey, barely seen). Insert in (*d*) illustrates the reference prey adaptive radiation showing that the width of the resource distribution tends to be less wide compared to predator–prey radiations. All the results presented are based on radiations with low prey niche width (*σ_α_* = 0.1) and other model parameters were set to: *u*_opt_ = 0; *K*_0_ = 10 000; *σ_K_* = 1; *r* = 1; *d* = 0.2; *c* = 0.3; *μ*_prey_ = 0.01.

Adaptive radiations of trophic communities show that predator–prey interactions largely interrupt prey diversification and we find a general negative relationship between prey diversification and predator niche width and efficiency (figure 4*a*–*c*). Nevertheless, predation can push the prey diversity beyond the diversity of the prey reference community when predator niche width is high and when prey niche width, predator efficiency, and mutation probability are low, especially in late stages of the radiation (figure 4*a* and electronic supplementary material, appendix 1, S1–S4). The reason for this interesting exception can be viewed in the radiations (figure 5). The predator interrupts the second and third branching such that they occur later in evolutionary time than they do in the reference community. They also occur further apart in trait space compared to the reference, as the predator pushes the prey into the peripheral parts of the resource distribution. Multiple distinct prey clades are then radiating in trait space, a wide niche space is filled up and a diverse community with a wide trait distribution will eventually emerge (figure 5*a*,*b*). More specifically the width of the resource distribution widens from −1.6 to 1.6 in the reference community to −2.5 to 2.4 in the predator–prey community.

The reason for the general decline in prey diversity owing to predation can be because of the effect that predators have on the disruptive selection on prey (figure 2) or because of the predator-induced decline in prey abundance (figure 4*g*–*i*). Predator diversity builds up with time (figure 4*d*–*f*), especially at intermediate predator niche width, leading to more predators that ultimately affects the prey radiation process. Furthermore, we identify additional macroevolutionary effects. Either the predator can co-evolve with the prey, radiate alongside the prey adaptive radiation (co-radiate) and continuously interrupt prey radiations; or alternatively, the predator can exclude the prey from parts of trait space and thus restrict prey radiation. Co-radiation mainly occurs at intermediate predator niche width (e.g. *σ_a_* = 0.3) and low to intermediate mutation probability (*μ*_pred_ = 0.005 and 0.01) (figure 5*a*). The ultimate effect of co-radiation will, however, also depend on predator efficiency. If efficiency is low (e.g. *b*_max_ = 0.0001, as figure 5*a*) the predators have little or no effect on prey communities even though the predator is co-radiating. But if efficiency is high (e.g. *b*_max_ = 0.0005) predators can leave gaps in the prey trait distribution (figure 5*b*) and thus reduce prey diversity. During such conditions, the widening effect of the prey trait distribution still occurs but this effect on prey diversity is counteracted by the predator-induced gaps in prey niche space. Conversely, predators excluding the prey from large parts of trait space without co-radiation, occurs when the predator's efficiency is large (e.g. *b*_max_ = 0.0007), niche width is low (e.g. *σ_a_* < 0.1) and mutation probability is low (e.g. *μ*_pred_ = 0.005) (figure 5*c*). The repelling force away from *u*_opt_ seen in equation (3.9) is strong and the system is pushed away from this point before the first branching occurs. The prey then radiates in parts of niche space where the predator is not present. The predator will not branch during such conditions, as there will be now disruptive selection on predators. Finally, we find a clear effect of predator mutation probability showing that high values (e.g. *μ*_pred_ = 0.1), in particular in combination with high predator efficiency (e.g. *b*_max_ = 0.0007), can interrupt the branching altogether (figure 5*d*).

## 6. Discussion

Ecological release through colonization of a novel habitat or mutations leading to innovations are arguably some of the most important prerequisites for rapid speciation and morphological diversification [2]. Many natural clades have diversified through such ecological release followed by diversification in adaptive radiations [2–4,18,41]. Competition, which may be the most prominent factor that affects adaptive radiations can both promote and interrupt diversification [42]. Similarly, predation has been suggested to promote divergent selection in prey [19,20,43]. Here we conclude that intraspecific competition is crucial for diversification, but diversification rate slows down as interspecific competition increases. Furthermore, predation generally has a negative effect on prey diversification through decreased population sizes, decreased disruptive selection and through the exclusion of prey from parts of niche space.

More specifically, we identify a clear relationship between niche width of the radiating organism, diversification rate and community diversity (figure 3*a*). The fact that niche width affects eco-evolutionary speciation is known from before [6,7] and here we confirm results from the adaptive dynamics literature that a narrow niche width also facilitates adaptive radiations. Similar to macroevolutionary patterns that have been statistically quantified by several empirical studies [33,34] we see a decrease in diversification rate with evolutionary time and the decrease is directly linked to the niche width of the radiating organism. The current knowledge of the mechanistic underpinning of such empirical patterns is limited, but by combining knowledge from micro-evolutionary theory and our simulations we identify three processes that combine to produce such diversity dependent patterns. First, intraspecific competition is crucial for each branching event as this is the main driver of diversification. Second, as diversity increases, niche availability is decreased owing to interspecific competition. The fitness landscape becomes more shallow, and the speed of evolution (formulated in the last term of equation (3.4)) is thus also reduced. Third, a general decrease in population size also reduces evolvability (related to N* in equation (3.4)) and thus slows down diversification. All the proposed effects of competition listed in the introduction of this paper are thus acting and interacting in the same adaptive radiation only the relative strength of them change as the radiation progress.

We show novel results on how predators tend to decrease prey diversification (figures 2 and 4). Predation can decrease competition driven disruptive selection in prey and predation also decreases prey abundance (figure 4*g*–*i*) which lowers the speed of prey diversification. In line with micro-evolutionary theory and previous experimental work [4,36] we thus conclude that that predator niche width, attack efficiency, and mutation probability will affect prey radiations by altering the prey fitness landscape and prey population sizes. Interestingly, we also find that predators can exclude the prey from parts of trait space and thus restrict prey diversification. Finally, even though we show that predation can increase disruptive selection on prey species (figure 2), we rarely see an increased diversification rate in co-evolving communities compared to competitive communities only. Predators can promote prey speciation in our simulations, but if this occurs early in the adaptive radiation predators tend to go extinct after the first prey branching (when predator niche width was low) or not branch (when predator niche width was high). Predator-driven adaptive radiations [44] thus seem difficult, at least under the assumptions tested with our model. Nevertheless, we do see that predator–prey co-evolution can induce high prey diversity compared to the reference competitive community, especially in late phases of the radiations and when predator efficiency is low, and predator mutation probability is low to intermediate (electronic supplementary material, appendix 1 and figures S1–S4). This unprecedented pattern is because of an interesting phenomenon of predators pushing prey communities to evolve into a wide niche space and thus increase the width of their trait distribution and diversity that, to our knowledge, has not been observed or suggested before.

The results discussed above facilitate a general understanding of the eco-evolutionary drivers of adaptive radiations through the link between microevolutionary processes (equations (3.3)–(3.10)) and macroevolutionary patterns called for by Gavrilets [26]. Although equations (3.3)–(3.10) do not directly apply to macro-evolutionary processes and the speed of adaptive radiations, we use them as guidance in our investigations and interpretation of large-scale community patterns. We thus provide a quantitative link between the concept of ecological release through innovation, eco-evolutionary speciation and adaptive radiations reviewed by Yoder, Clancey [2]. Our investigations give mechanistic insights to density and frequency dependent speciation, and the role of competition in macroevolution called for by [33,42] and the largely unknown effect that predation may have on prey adaptive radiations [4,45]. That said, as in any modelling study, our results are restricted by model assumptions and the parameter space analysed. We model asexual organisms, we assume a constant environment and resource availability, we assume a one-dimensional trait space, we omit space, and we make explicit assumptions about competition, resource utilization through trait matching and linear predator functional response (initial simulations suggest that exploring the influence of a type 3 functional response would be very interesting). It may also seem unrealistic to compare communities that contain species with either a narrow or a broad niche width, as most communities will have both specialist and generalist species. Niche width can also change with evolutionary time [46,47] and it can be argued that our community assembly model through ecological speciation is unrealistic as few if any natural communities are assembled purely by an adaptive radiation. Even though our results may be directly applicable to non-sexual organisms, our aim is, however, not to model any particular empirical system but rather investigate the fundamental causal effects between ecological opportunity, eco-evolutionary microevolutionary processes, and adaptive radiations. We thus isolate processes by specifying an ecological model and simulate the eco-evolutionary assembly processes. We base this simulation approach on a well-established ecological trait-based modelling approach and eco-evolutionary adaptive dynamics theory. We do not assume speciation rates or community richness. Instead, our minimal assumptions about traits, trait matching and trait evolution drive the eco-evolutionary dynamics, and the community patterns will be emergent properties of those focused assumptions. We elucidate some of the mechanisms that underpin adaptive radiations, and it will be intriguing to see future studies that may attempt to expand and apply this theoretical investigation.

## Supplementary Material

Appendix 1

## Data accessibility

This article has no additional data.

## References

[1] Ellner SP, Geber MA, Hairston NG 2011 Does rapid evolution matter? Measuring the rate of contemporary evolution and its impacts on ecological dynamics. Ecol. Lett. 14, 603–614. (10.1111/j.1461-0248.2011.01616.x) 21518209

[2] Yoder JB et al. 2010 Ecological opportunity and the origin of adaptive radiations. J. Evol. Biol. 23, 1581–1596. (10.1111/j.1420-9101.2010.02029.x) 20561138

[3] Losos JB 2010 Adaptive radiation, ecological opportunity, and evolutionary determinism. Am. Nat. 175, 623–639. (10.1086/652433) 20412015

[4] Meyer JR, Kassen R 2007 The effects of competition and predation on diversification in a model adaptive radiation. Nature 446, 432–435. (10.1038/nature05599) 17377581

[5] Stroud JT, Losos JB 2016 Ecological opportunity and adaptive radiation. Ann. Rev. Ecol. Evol. Syst. 47, 507–532. (10.1146/annurev-ecolsys-121415-032254)

[6] Geritz SAH, Kisdi E, Meszena G, Metz JAJ 1998 Evolutionarily singular strategies and the adaptive growth and branching of the evolutionary tree. Evol. Ecol. 12, 35–57. (10.1023/A:1006554906681)

[7] Dieckmann U, Doebeli M 1999 On the origin of species by sympatric speciation. Nature 400, 354–357. (10.1038/22521) 10432112

[8] Brännström Å, Johansson J, von Festenberg N 2013 The hitchhiker's guide to adaptive dynamics. Games 4, 304–328. (10.3390/g4030304)

[9] Pontarp M, Ripa J, Lundberg P 2012 On the origin of phylogenetic structure in competitive metacommunities. Evol. Ecol. Res. 14, 269–284.

[10] Pontarp M, Ripa J, Lundberg P 2015 The biogeography of adaptive radiations and the geographic overlap of sister species. Am. Nat. 186, 565–581. (10.1086/683260) 26655771

[11] Pontarp M, Wiens JJ 2016 The origin of species richness patterns along environmental gradients: uniting explanations based on time, diversification rate, and carrying capacity. J. Biogeogr. 44, 722–735. (10.1111/jbi.12896)

[12] Schluter D 2000 The ecology of adaptive radiations. Oxford, UK: Oxford University Press.

[13] Grant PR 1998 Evolution on islands. Oxford, UK: Oxford University Press.

[14] MacArthur HR, Wilson EO 1968 The theory of island biogeography. Princeton, NJ: Princeton University Press.

[15] Kisel Y, McInnes L, Toomey NH, Orme CDL 2011 How diversification rates and diversity limits combine to create large-scale species-area relationships. Phil. Trans. R. Soc. B 366, 2514–2525. (10.1098/rstb.2011.0022) 21807732 PMC3138612

[16] Stevens MHH, Sanchez M, Lee J, Finkel SE 2007 Diversification rates increase with population size and resource concentration in an unstructured habitat. Genetics 177, 2243–2250. (10.1534/genetics.107.076869) 18073429 PMC2219503

[17] Bailey SF, Dettman JR, Rainey PB, Kassen R 2016 Correction to ‘Competition both drives and impedes diversification in a model adaptive radiation’ (vol. 280, 20131253, 2013). Proc. R. Soc. B 283, ARTN 20160763 (10.1098/rspb.2016.0763) PMC3730596 23843392

[18] Tan JQ, Slattery MR, Yang X, Jiang L 2016 Phylogenetic context determines the role of competition in adaptive radiation. Proc. R. Soc. B 283, ARTN 20160241 (10.1098/rspb.2016.0241) PMC4936025 27335414

[19] Allen WL, Baddeley R, Scott-Samuel NE, Cuthill IC 2013 The evolution and function of pattern diversity in snakes. Behav. Ecol. 24, 1237–1250. (10.1093/beheco/art058)

[20] Langerhans RB 2009 Trade-off between steady and unsteady swimming underlies predator-driven divergence in *Gambusia affinis*. J. Evol. Biol. 22, 1057–1075. (10.1111/j.1420-9101.2009.01716.x) 21462405

[21] Brown JS, Vincent TL 1992 Organization of predator-prey communities as an evolutionary game. Evolution 46, 1269–1283. (10.2307/2409936) 28569003

[22] Ripa J, Storlind L, Lundberg P, Brown JS 2009 Niche co-evolution in consumer-resource communities. Evol. Ecol. Res. 11, 305–323.

[23] Ito H, Shimada M, Ikegami T 2009 Coevolutionary dynamics of adaptive radiation for food-web development. Popul. Ecol. 51, 65–81. (10.1007/s10144-008-0113-5)

[24] Anderson CM, Langerhans RB 2015 Origins of female genital diversity: predation risk and lock-and-key explain rapid divergence during an adaptive radiation. Evolution 69, 2452–2467. (10.1111/evo.12748) 26259062

[25] Vamosi SM 2005 On the role of enemies in divergence and diversification of prey: a review and synthesis. Can. J. Zool. 83, 894–910. (10.1139/Z05-063)

[26] Gavrilets S 2014 Models of speciation: where are we now? J. Hered. 105, 743–755. (10.1093/jhered/esu045) 25149251

[27] Brännström A, Loeuille N, Loreau M, Dieckmann U 2011 Emergence and maintenance of biodiversity in an evolutionary food-web model. Theor. Ecol. 4, 467–478. (10.1007/s12080-010-0089-6)

[28] Sauterey B, Ward B, Rault J, Bowler C, Claessen D 2017 The implications of eco-evolutionary processes for the emergence of marine plankton community biogeography. Am. Nat. 190, 116–130. (10.1086/692067) 28617645

[29] Doebeli M, Dieckmann U 2003 Speciation along environmental gradients. Nature 421, 259–264. (10.1038/Nature01274) 12529641

[30] Heinz SK, Mazzucco R, Dieckmann U 2009 Speciation and the evolution of dispersal along environmental gradients. Evol. Ecol. 23, 53–70. (10.1007/s10682-008-9251-7)

[31] Ito HC, Dieckmann U 2007 A new mechanism for recurrent adaptive radiations. Am. Nat. 170, E96–E111. (10.1086/521229) 17891728

[32] Claessen D, Andersson J, Persson L, de Roos AM 2007 Delayed evolutionary branching in small populations. Evol. Ecol. Res. 9, 51–69.

[33] Rabosky DL 2013 Diversity-dependence, ecological speciation, and the role of competition in macroevolution. Ann. Rev. Ecol. Evol. Syst. 44, 481–502. (10.1146/annurev-ecolsys-110512-135800)

[34] Price TD et al. 2014 Niche filling slows the diversification of Himalayan songbirds. Nature 509, 222–225. (10.1038/nature13272) 24776798

[35] Metz JAJ 2011 Thoughts on the geometry of meso-evolution: collecting mathematical elements for a postmodern synthesis. In The mathematics of Darwin's Legacy (eds Chalub FACC, Rodrigues JR), pp. 193–231. Basel, Switzerland: Springer.

[36] Doebeli M, Dieckmann U 2000 Evolutionary branching and sympatric speciation caused by different types of ecological interactions. Am. Nat. 156, S77–S101. (10.1086/303417) 29592583

[37] Case TJ 2000 An illustrated guide to theoretical ecology. Oxford, UK: Oxford University Press.

[38] Pontarp M, Petchey OL 2016 Community trait overdispersion due to trophic interactions: concerns for assembly process inference. Proc. R. Soc. B 283, 20161602 (10.1098/rspb.2016.1602) PMC5069517 27733548

[39] Metz JAJ, Nisbet RM, Geritz SAH 1992 How should we define fitness for general ecological scenarios. Trends Ecol. Evol. 7, 198–202. (10.1016/0169-5347(92)90073-k) 21236007

[40] Dieckmann U, Law R 1996 The dynamical theory of coevolution: a derivation from stochastic ecological processes. J. Math. Biol. 34, 579–612. (10.1007/Bf02409751) 8691086

[41] Hughes CE, Atchison GW 2015 The ubiquity of alpine plant radiations: from the Andes to the Hengduan Mountains. New Phytol. 207, 275–282. (10.1111/nph.13230) 25605002

[42] Bailey SF, Dettman JR, Rainey PB, Kassen R 2013 Competition both drives and impedes diversification in a model adaptive radiation. Proc. R. Soc. B 280, ARTN 20131253 (10.1098/rspb.2013.1253) PMC3730596 23843392

[43] Broeckhoven C, Diedericks G, Mouton PLN 2015 What doesn't kill you might make you stronger: functional basis for variation in body armour. J. Anim. Ecol. 84, 1213–1221. (10.1111/1365-2656.12414) 26104546

[44] Nosil P, Crespi BJ 2006 Experimental evidence that predation promotes divergence in adaptive radiation. Proc. Natl Acad. Sci. USA 103, 9090–9095. (10.1073/pnas.0601575103) 16754870 PMC1482571

[45] Yoder JB, Nuismer SL 2010 When does coevolution promote diversification? Am. Nat. 176, 802–817. (10.1086/657048) 20950142

[46] Gillespie RG 2016 Island time and the interplay between ecology and evolution in species diversification. Evol. Appl. 9, 53–73. (10.1111/eva.12302) 27087839 PMC4780372

[47] Ackermann M, Doebeli M 2004 Evolution of niche width and adaptive diversification. Evolution 58, 2599–2612. (10.1111/j.0014-3820.2004.tb01614.x) 15696740

