# Supplementary material for: Ecological opportunity and predator–prey interactions: linking eco-evolutionary processes and diversification in adaptive radiations
Source: Proc Biol Sci. 2018 Mar 7;285(1874):20172550. doi: 10.1098/rspb.2017.2550 (PMC5879621; doi:10.1098/rspb.2017.2550)
Supplement: Appendix 1 [file rspb20172550supp1.pdf]

Proceedings of the Royal Society B

SUPPORTING INFORMATION

Ecological opportunity and predator-prey interactions: linking eco-evolutionary processes and diversification in adaptive radiations

Mikael Pontarp[1,2] and Owen L Petchey[1]

[1]Department of Evolutionary Biology and Environmental Studies, University of Zurich, Winterthurerstrasse 190, 8057 Zurich, Switzerland

[2] Department of Ecology and Environmental Science, Umeå University, 90187 Umeå, Sweden

Article DOI: 10.1098/rspb.[paper ID in form xxxx.xxxx e.g. 10.1098/rspb.2016.0049]

Appendix 1

Robustness

All the results presented in the main paper are based on radiations with low prey niche width (σ_α = 0.1) but quantitatively robust also for wider prey niche width (Appendix 1, figures. S1-S12) and longer running (5000 evolutionary steps, see examples in figure S13-S15) simulations. As an additional robustness check, we also simulated radiations with 25% increase in both parameters *r* and *d*, a 25 % decrease in *r* and *d*, and a 25% increase in *r* and a 25 % decrease in *d* (figures S13-S15). We also inspected the ecological dynamics throughout the radiations and we see no sign of unstable ecological dynamics throughout our simulations. Our conclusions presented in the main paper thus remain the same.

The simulations are largely deterministic (with some stochastic elements included by random mutations). We see low variation in prey diversity across 10 replicates, standard deviation < 2 for all σ_α. Variation increases slightly, plausibly due to fewer (5) replicates and increased community complexity, in the predator-prey simulations. Standard deviation (std) in prey diversity across replicates in predator-prey simulations is low (std < 3) when bmax = 0.0001 and then increases when bmax is increased to 0.0003 (std < 6) and 0.0005 (std < 9). This increase in variation is however mainly due to increased variation in simulations with low predator niche width (σ_a =0.1), due to predator extinctions in some simulations. Std in predator diversity were >3 for simulations where σ_a =0.3-0.7.

Figure legends

Figure S1-S4

Predator-prey community adaptive radiation data. Prey diversity as a function of time and predator niche width (range 0.1(black) – 0.7 (light gray)) for a range of predator mutation

probability and efficiency. Dashed line represent a prey reference community and solid lines denotes predator-prey community data. See panel titles for predator parameter settings. Prey niche width (denoted as sig_a) range 0.1 (figure S1); 0.3 (figure S2); 0.5 (figure S3) 0.7 (figure S4).

Figure S5-S8

Predator-prey community adaptive radiation data. Predator diversity as a function of time and predator niche width (range 0.1(black) – 0.7 (light gray)) for a range of predator mutation probability and efficiency. See panel titles for predator parameter settings. Prey niche width (denoted as sig_a) range 0.1 (figure S5); 0.3 (figure S6); 0.5 (figure S7) 0.7 (figure S8).

Figure S9-S12

Predator-prey community adaptive radiation data. Mean prey abundance as a function of time and predator niche width (range 0.1(black) – 0.7 (light gray)) for a range of predator mutation probability and efficiency. Dashed line represent a prey reference community and solid lines denotes predator-prey community data. See panel titles for predator parameter settings. Prey niche width (denoted as sig_a) range 0.1 (figure S9); 0.3 (figure S10); 0.5 (figure S11) 0.7 (figure S12).

Figure S13-S15

Predator-prey adaptive radiations across parameter space for different prey growth (r) and (d) values. Fig. S13 shows results where r = 0.75 and d = 0.15. Fig. S14 shows results where r = 1.25 and d = 0.25. Fig. S15 shows results where r = 1.25 and d = 0.15. The general patterns are similar among these parameter combinations. Co-radiation at intermediate predator niche

width (σ_a = 0.3), low predator mutation probability (μ_{pred} = 0.005) and low (a) and high (b) predator efficiency (b_{max} = 0.0001 (a) and 0.0005 (b)). Predators excluding the prey from parts of trait space when the predator's efficiency is large (b_{max} = 0.0007), niche width is low (e.g. σ_a < 0.1) and mutation probability is low (μ_{pred} = 0.005) (c). Note that the prey can escape the excluding affect induced by the predator if a prey mutant invades on the opposite side of the predator trait (panel c in Fig S13) or if the predator goes extinct (panel c in Fig. S15). High values of predator mutation probability (μ_{pred} = 0.1), in combination with high predator efficiency (b_{max} = 0.0007) interrupts the branching all together, only one predator and one prey population co-evolve in trait space with the predator trait (red) completely overlapping the prey (gray, barely seen) (d). All the results presented is based on radiations with low prey niche width (σ_α = 0.1) and other model parameters were set to: u_{opt}=0; K_0=10000; σ_K =1; c = 0.3; μ_{prey} = 0.01.

Figures

Figure S1

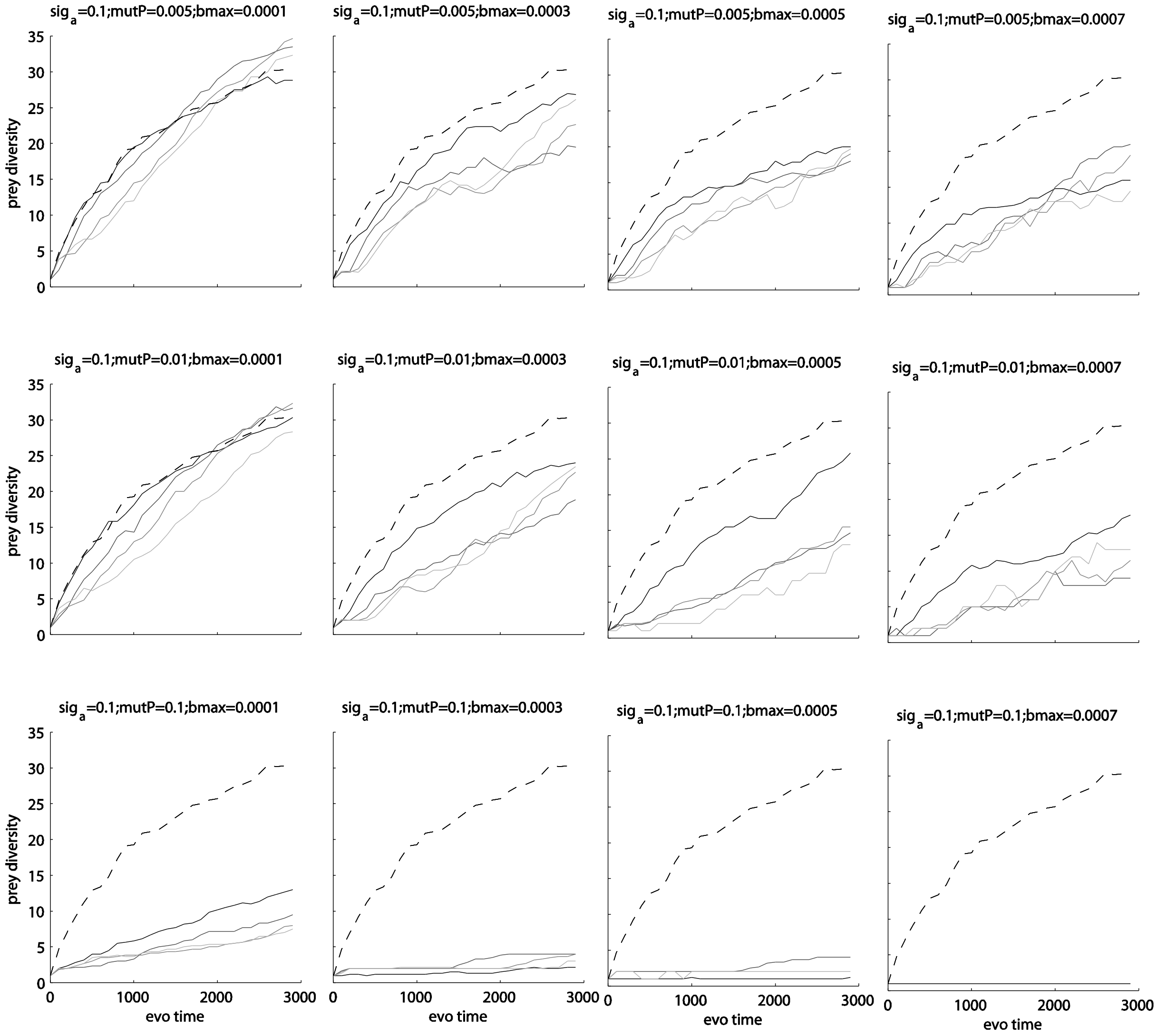

Figure S2

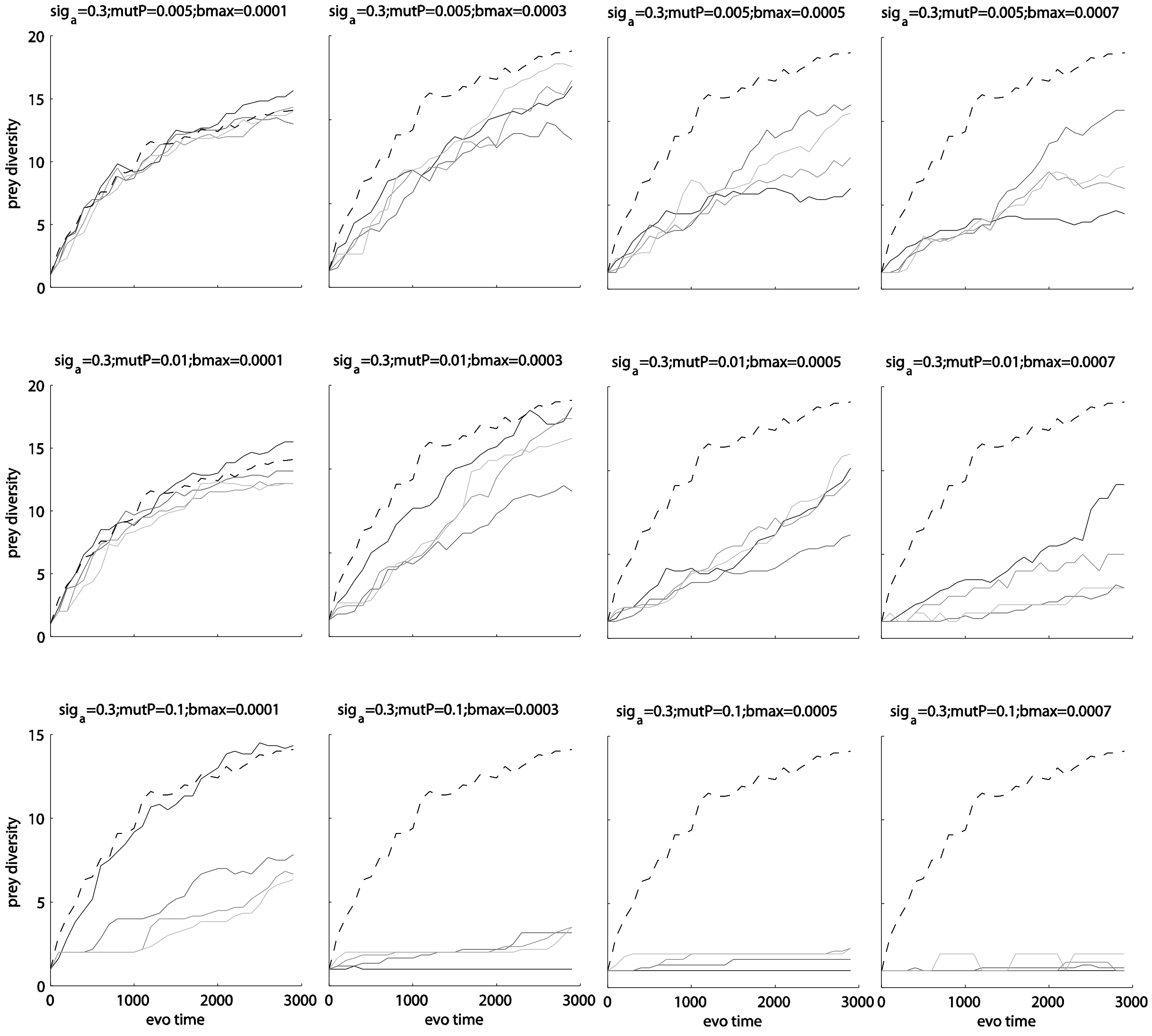
sig_a=0.3;mutP=0.005;bmax=0.0001
sig_a=0.3;mutP=0.005;bmax=0.0003
sig_a=0.3;mutP=0.005;bmax=0.0005
sig_a=0.3;mutP=0.005;bmax=0.0007
sig_a=0.3;mutP=0.01;bmax=0.0001
sig_a=0.3;mutP=0.01;bmax=0.0003
sig_a=0.3;mutP=0.01;bmax=0.0005
sig_a=0.3;mutP=0.01;bmax=0.0007
sig_a=0.3;mutP=0.1;bmax=0.0001
sig_a=0.3;mutP=0.1;bmax=0.0003
sig_a=0.3;mutP=0.1;bmax=0.0005
sig_a=0.3;mutP=0.1;bmax=0.0007
prey diversity
evo time
0
1000
2000
3000
0
5
10
15
20

Figure S3

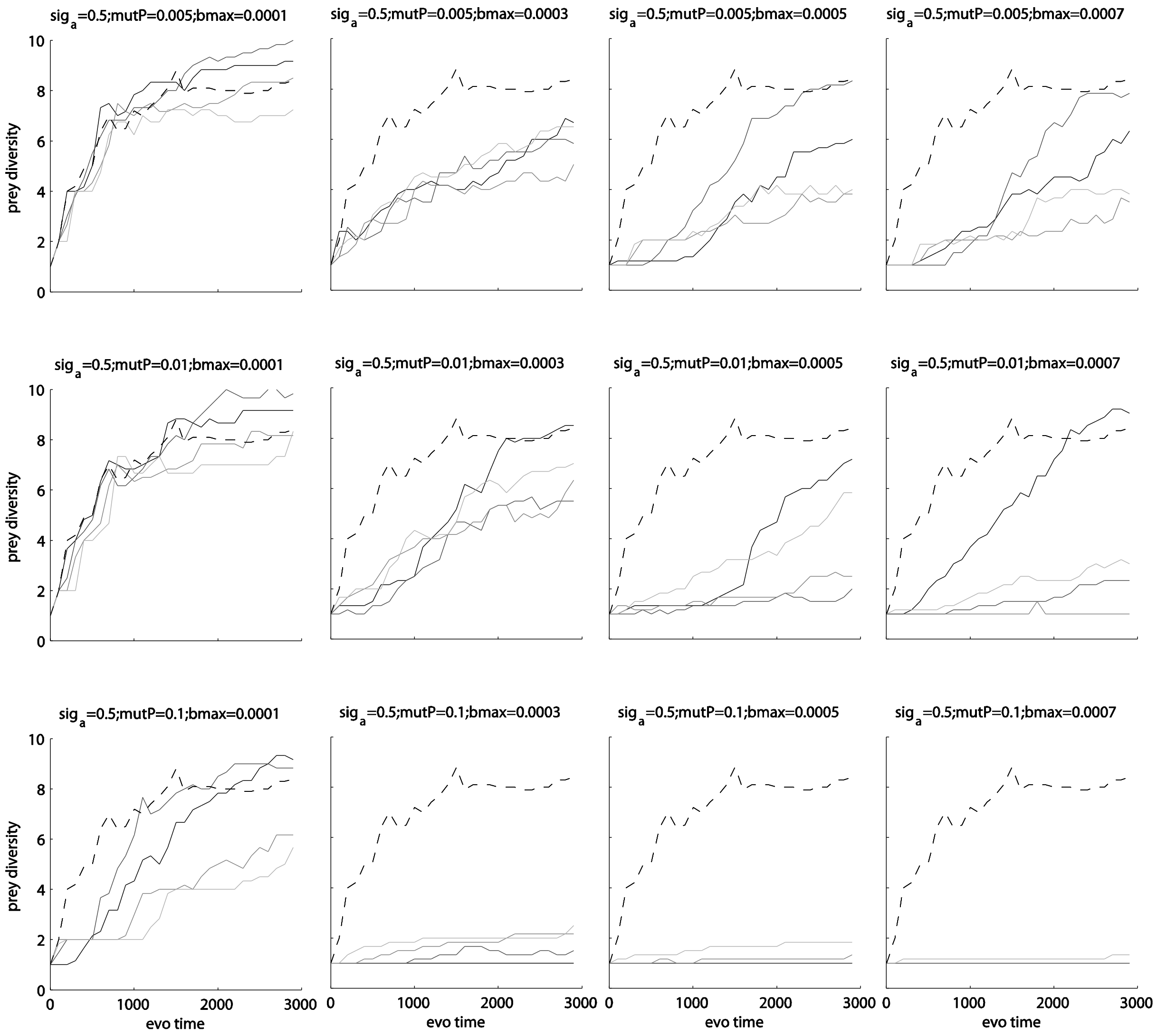

sig_a=0.5;mutP=0.005;bmax=0.0001
sig_a=0.5;mutP=0.005;bmax=0.0003
sig_a=0.5;mutP=0.005;bmax=0.0005
sig_a=0.5;mutP=0.005;bmax=0.0007
sig_a=0.5;mutP=0.01;bmax=0.0001
sig_a=0.5;mutP=0.01;bmax=0.0003
sig_a=0.5;mutP=0.01;bmax=0.0005
sig_a=0.5;mutP=0.01;bmax=0.0007
sig_a=0.5;mutP=0.1;bmax=0.0001
sig_a=0.5;mutP=0.1;bmax=0.0003
sig_a=0.5;mutP=0.1;bmax=0.0005
sig_a=0.5;mutP=0.1;bmax=0.0007
prey diversity
evo time
0
1000
2000
3000

Figure S4

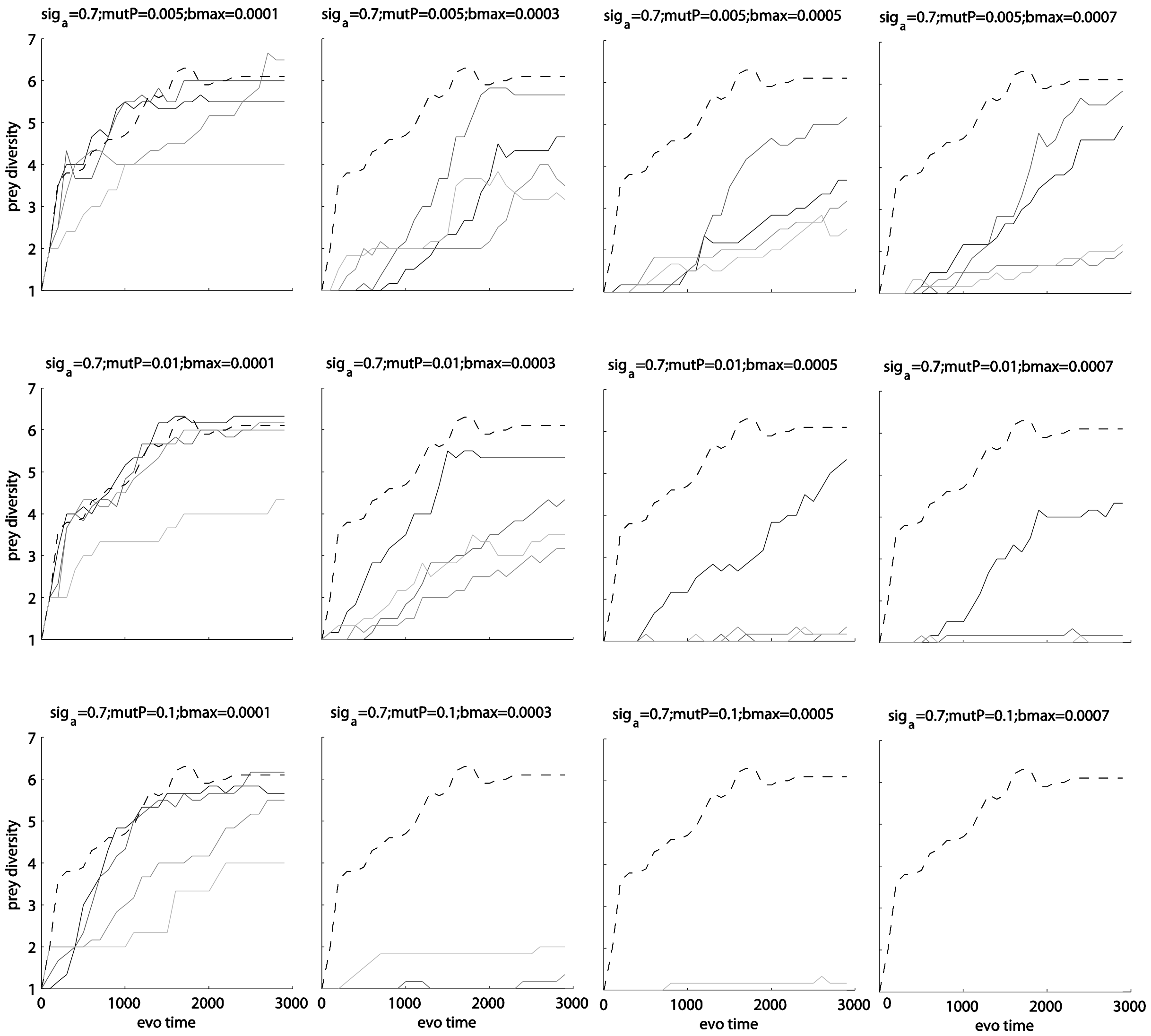
sig_a=0.7;mutP=0.005;bmax=0.0001
sig_a=0.7;mutP=0.005;bmax=0.0003
sig_a=0.7;mutP=0.005;bmax=0.0005
sig_a=0.7;mutP=0.005;bmax=0.0007
sig_a=0.7;mutP=0.01;bmax=0.0001
sig_a=0.7;mutP=0.01;bmax=0.0003
sig_a=0.7;mutP=0.01;bmax=0.0005
sig_a=0.7;mutP=0.01;bmax=0.0007
sig_a=0.7;mutP=0.1;bmax=0.0001
sig_a=0.7;mutP=0.1;bmax=0.0003
sig_a=0.7;mutP=0.1;bmax=0.0005
sig_a=0.7;mutP=0.1;bmax=0.0007
prey diversity
evo time
1
2
3
4
5
6
7
0
1000
2000
3000

Figure S5

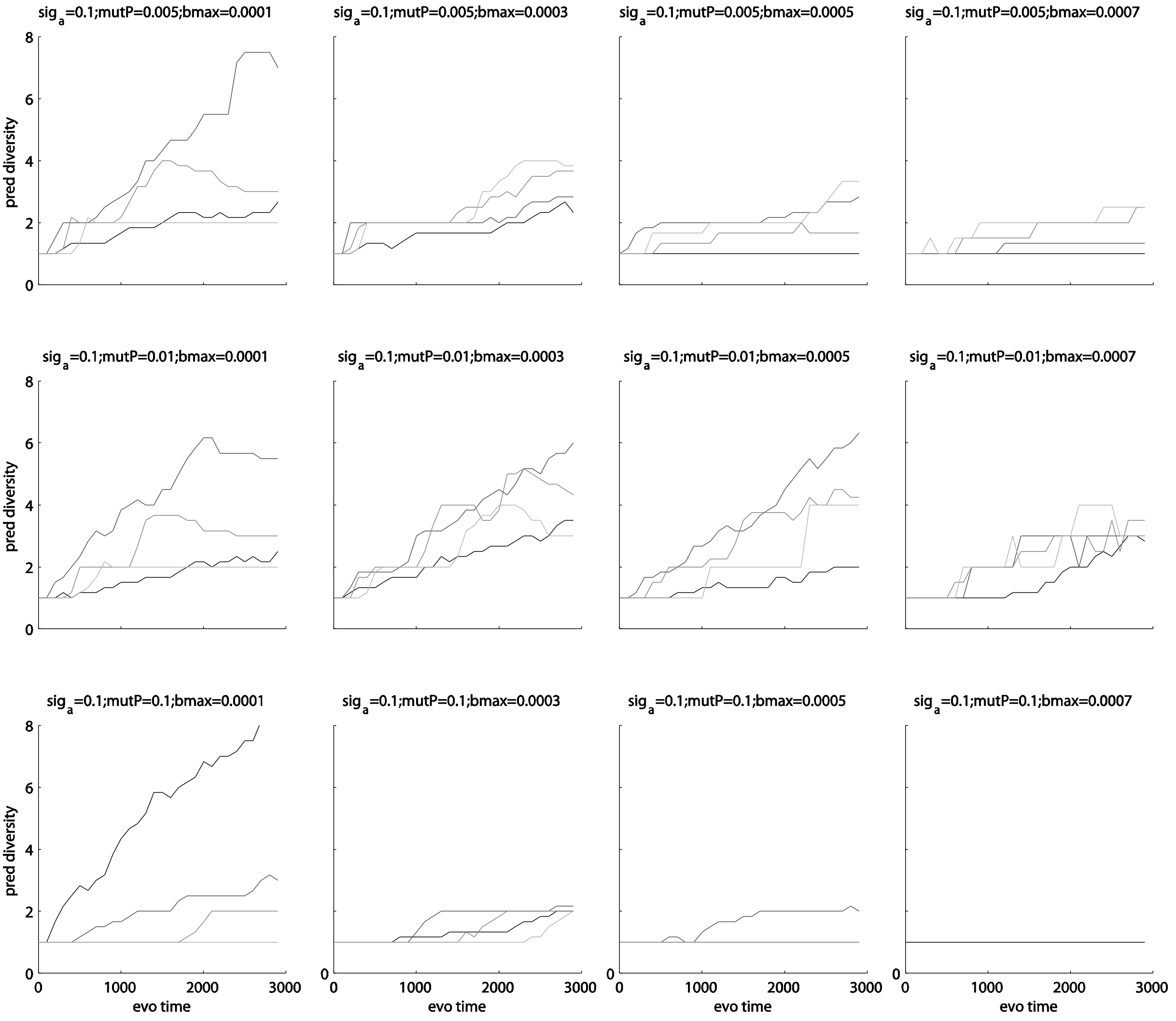
siga=0.1;mutP=0.005;bmax=0.0001
siga=0.1;mutP=0.005;bmax=0.0003
siga=0.1;mutP=0.005;bmax=0.0005
siga=0.1;mutP=0.005;bmax=0.0007
siga=0.1;mutP=0.01;bmax=0.0001
siga=0.1;mutP=0.01;bmax=0.0003
siga=0.1;mutP=0.01;bmax=0.0005
siga=0.1;mutP=0.01;bmax=0.0007
siga=0.1;mutP=0.1;bmax=0.0001
siga=0.1;mutP=0.1;bmax=0.0003
siga=0.1;mutP=0.1;bmax=0.0005
siga=0.1;mutP=0.1;bmax=0.0007
pred diversity
evo time
0
2
4
6
8
0
1000
2000
3000

Figure S6

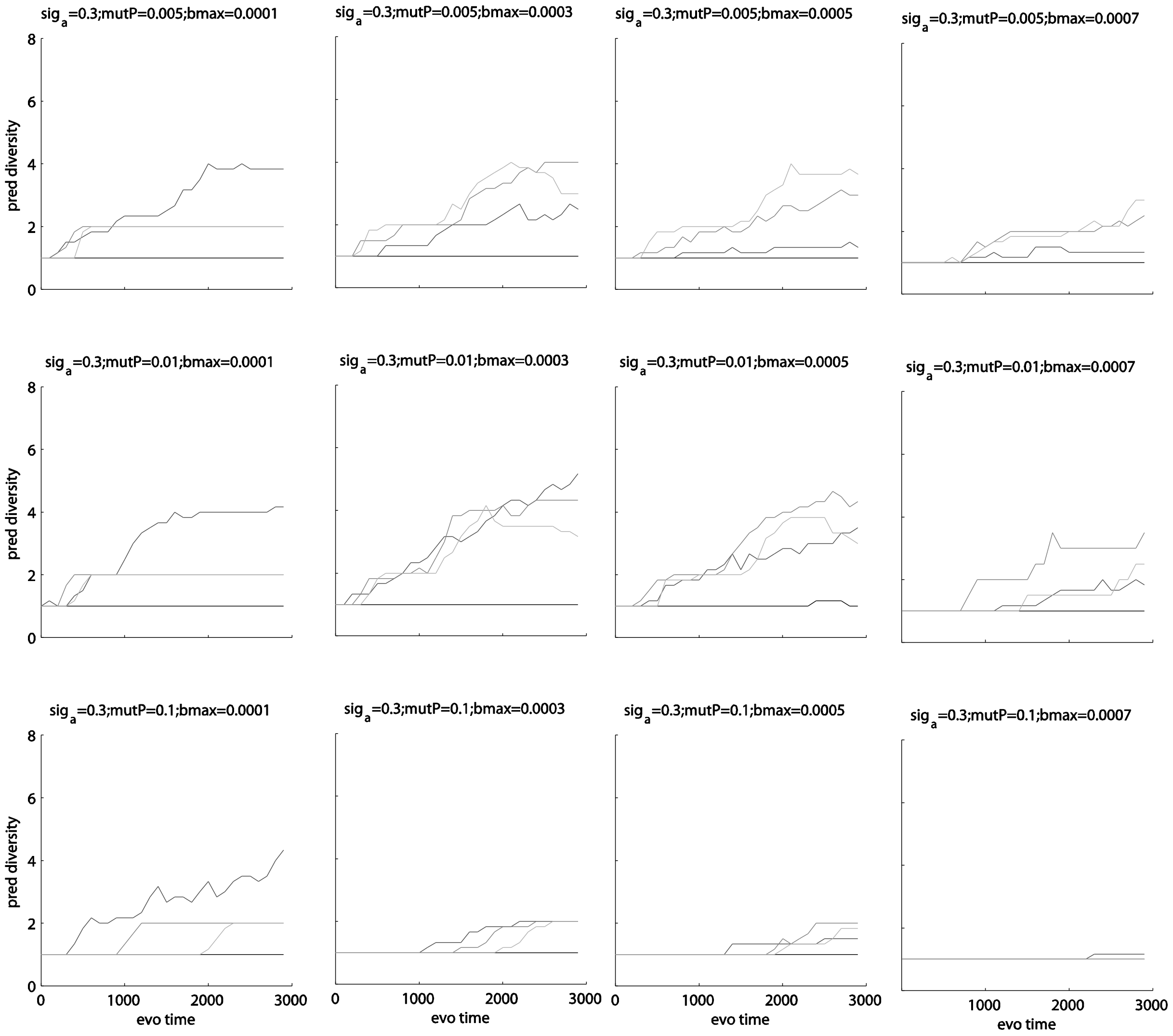
sig$_a$=0.3;mutP=0.005;bmax=0.0001
sig$_a$=0.3;mutP=0.005;bmax=0.0003
sig$_a$=0.3;mutP=0.005;bmax=0.0005
sig$_a$=0.3;mutP=0.005;bmax=0.0007
sig$_a$=0.3;mutP=0.01;bmax=0.0001
sig$_a$=0.3;mutP=0.01;bmax=0.0003
sig$_a$=0.3;mutP=0.01;bmax=0.0005
sig$_a$=0.3;mutP=0.01;bmax=0.0007
sig$_a$=0.3;mutP=0.1;bmax=0.0001
sig$_a$=0.3;mutP=0.1;bmax=0.0003
sig$_a$=0.3;mutP=0.1;bmax=0.0005
sig$_a$=0.3;mutP=0.1;bmax=0.0007
pred diversity
0
2
4
6
8
evo time
0
1000
2000
3000

Figure S7

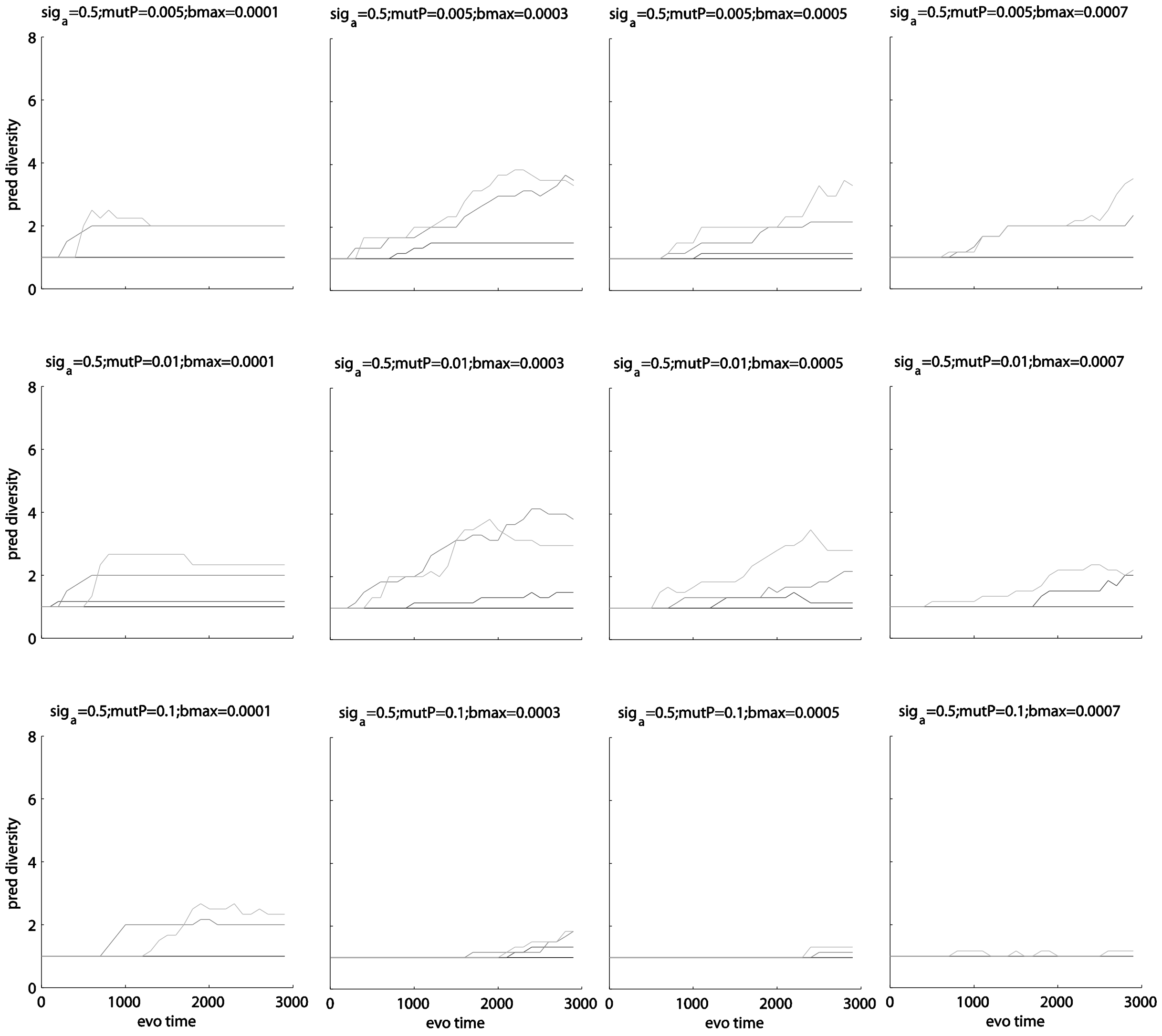
sig a=0.5;mutP=0.005;bmax=0.0001
sig a=0.5;mutP=0.005;bmax=0.0003
sig a=0.5;mutP=0.005;bmax=0.0005
sig a=0.5;mutP=0.005;bmax=0.0007
sig a=0.5;mutP=0.01;bmax=0.0001
sig a=0.5;mutP=0.01;bmax=0.0003
sig a=0.5;mutP=0.01;bmax=0.0005
sig a=0.5;mutP=0.01;bmax=0.0007
sig a=0.5;mutP=0.1;bmax=0.0001
sig a=0.5;mutP=0.1;bmax=0.0003
sig a=0.5;mutP=0.1;bmax=0.0005
sig a=0.5;mutP=0.1;bmax=0.0007
pred diversity
evo time
0
2
4
6
8
1000
2000
3000

Figure S8

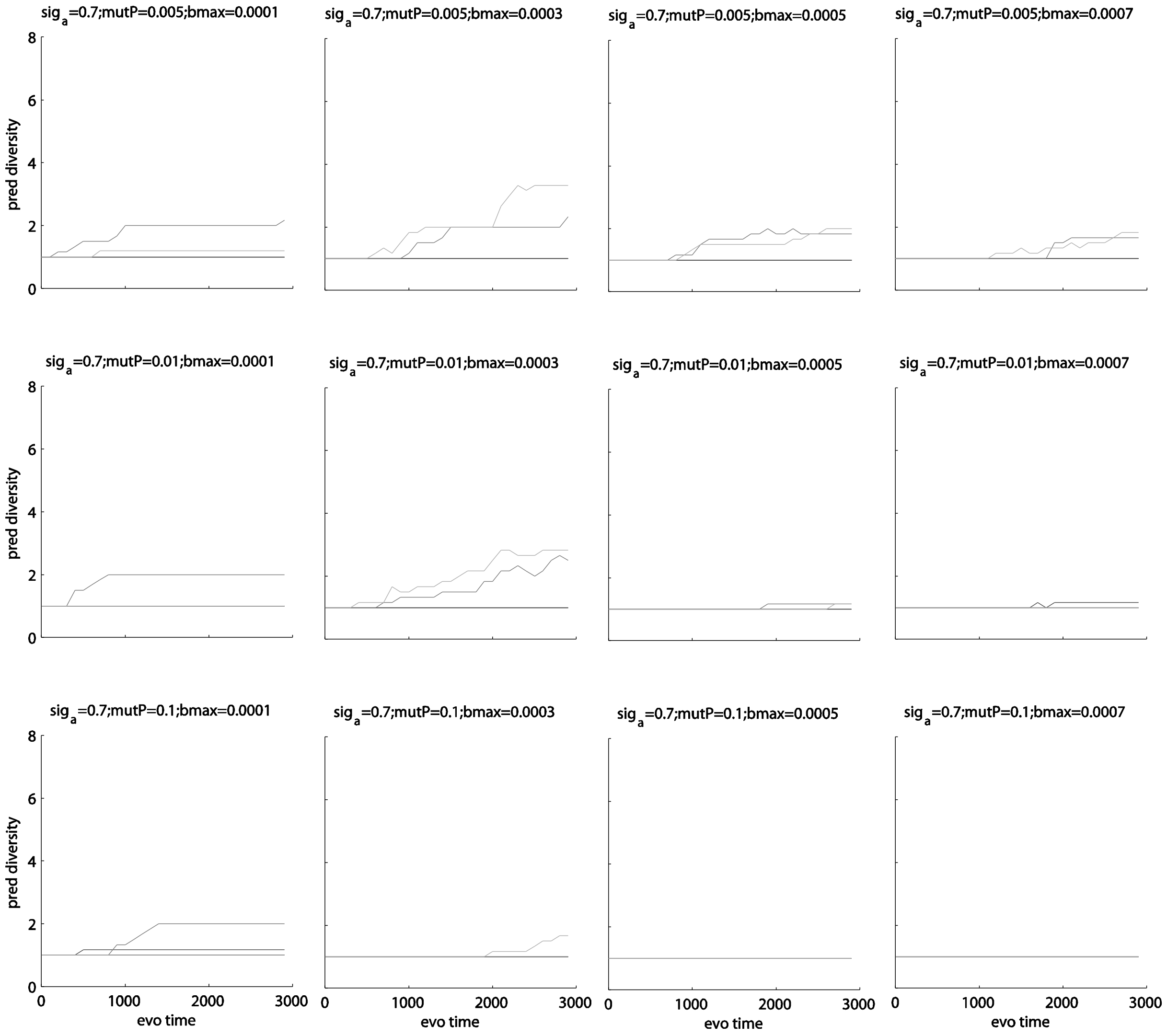
sig a=0.7;mutP=0.005;bmax=0.0001
sig a=0.7;mutP=0.005;bmax=0.0003
sig a=0.7;mutP=0.005;bmax=0.0005
sig a=0.7;mutP=0.005;bmax=0.0007
sig a=0.7;mutP=0.01;bmax=0.0001
sig a=0.7;mutP=0.01;bmax=0.0003
sig a=0.7;mutP=0.01;bmax=0.0005
sig a=0.7;mutP=0.01;bmax=0.0007
sig a=0.7;mutP=0.1;bmax=0.0001
sig a=0.7;mutP=0.1;bmax=0.0003
sig a=0.7;mutP=0.1;bmax=0.0005
sig a=0.7;mutP=0.1;bmax=0.0007
pred diversity
evo time
0
2
4
6
8
1000
2000
3000

Figure S9

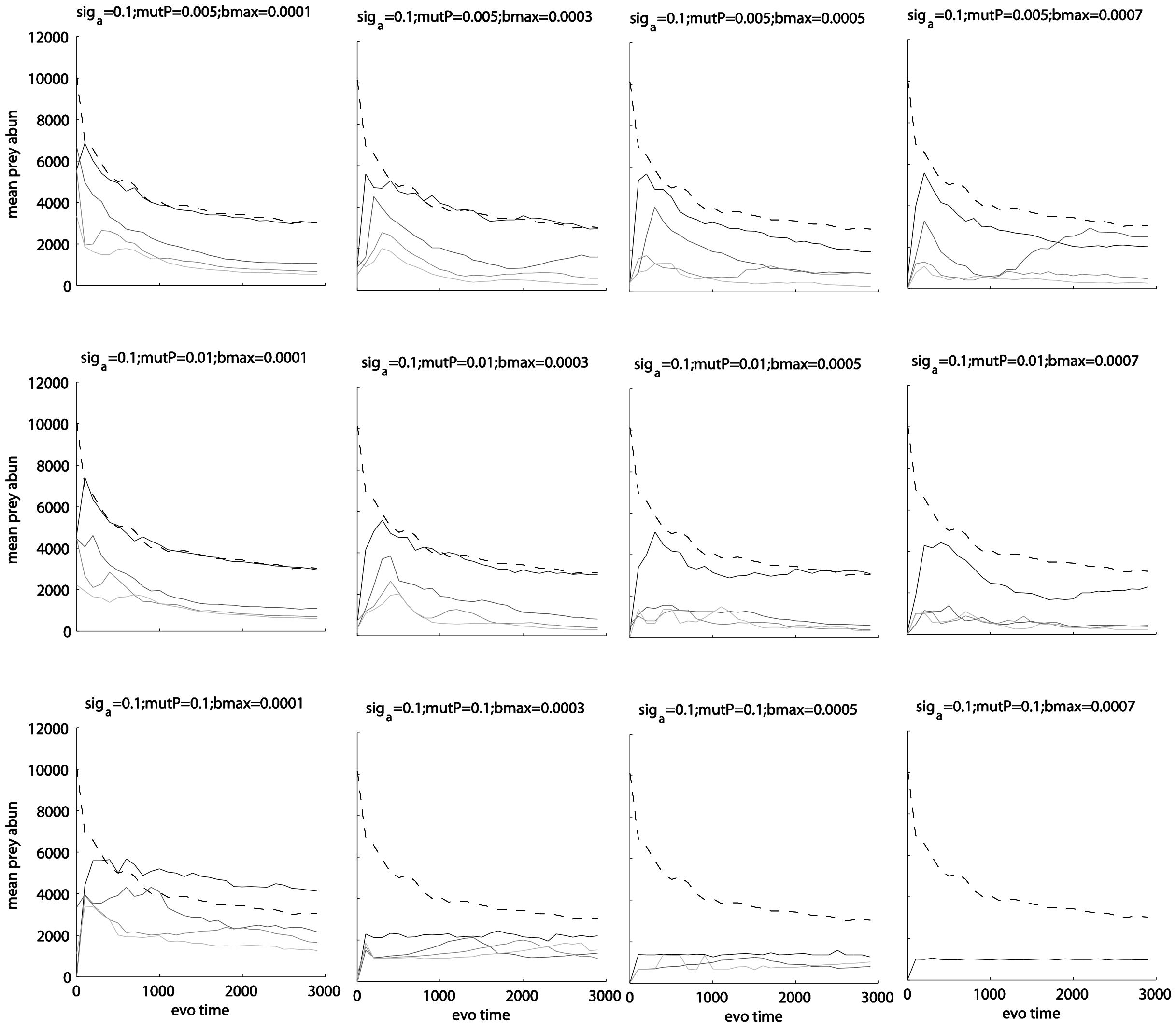
sig a=0.1;mutP=0.005;bmax=0.0001
sig a=0.1;mutP=0.005;bmax=0.0003
sig a=0.1;mutP=0.005;bmax=0.0005
sig a=0.1;mutP=0.005;bmax=0.0007
sig a=0.1;mutP=0.01;bmax=0.0001
sig a=0.1;mutP=0.01;bmax=0.0003
sig a=0.1;mutP=0.01;bmax=0.0005
sig a=0.1;mutP=0.01;bmax=0.0007
sig a=0.1;mutP=0.1;bmax=0.0001
sig a=0.1;mutP=0.1;bmax=0.0003
sig a=0.1;mutP=0.1;bmax=0.0005
sig a=0.1;mutP=0.1;bmax=0.0007
mean prey abun
0
2000
4000
6000
8000
10000
12000
evo time
0
1000
2000
3000

Figure S10

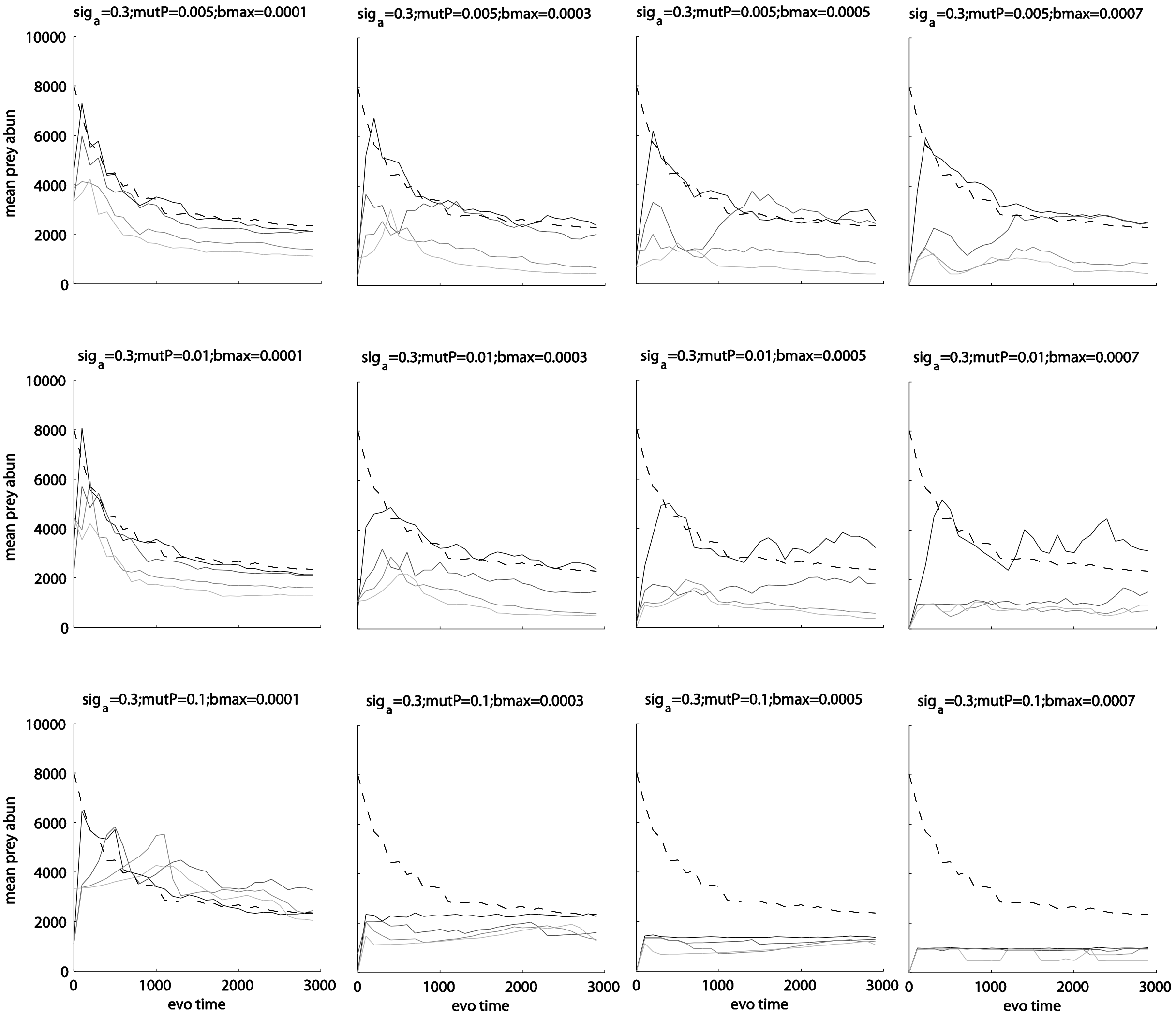
sig_a=0.3;mutP=0.005;bmax=0.0001
sig_a=0.3;mutP=0.005;bmax=0.0003
sig_a=0.3;mutP=0.005;bmax=0.0005
sig_a=0.3;mutP=0.005;bmax=0.0007
sig_a=0.3;mutP=0.01;bmax=0.0001
sig_a=0.3;mutP=0.01;bmax=0.0003
sig_a=0.3;mutP=0.01;bmax=0.0005
sig_a=0.3;mutP=0.01;bmax=0.0007
sig_a=0.3;mutP=0.1;bmax=0.0001
sig_a=0.3;mutP=0.1;bmax=0.0003
sig_a=0.3;mutP=0.1;bmax=0.0005
sig_a=0.3;mutP=0.1;bmax=0.0007
mean prey abun
evo time
0
2000
4000
6000
8000
10000
1000
3000

Figure S11

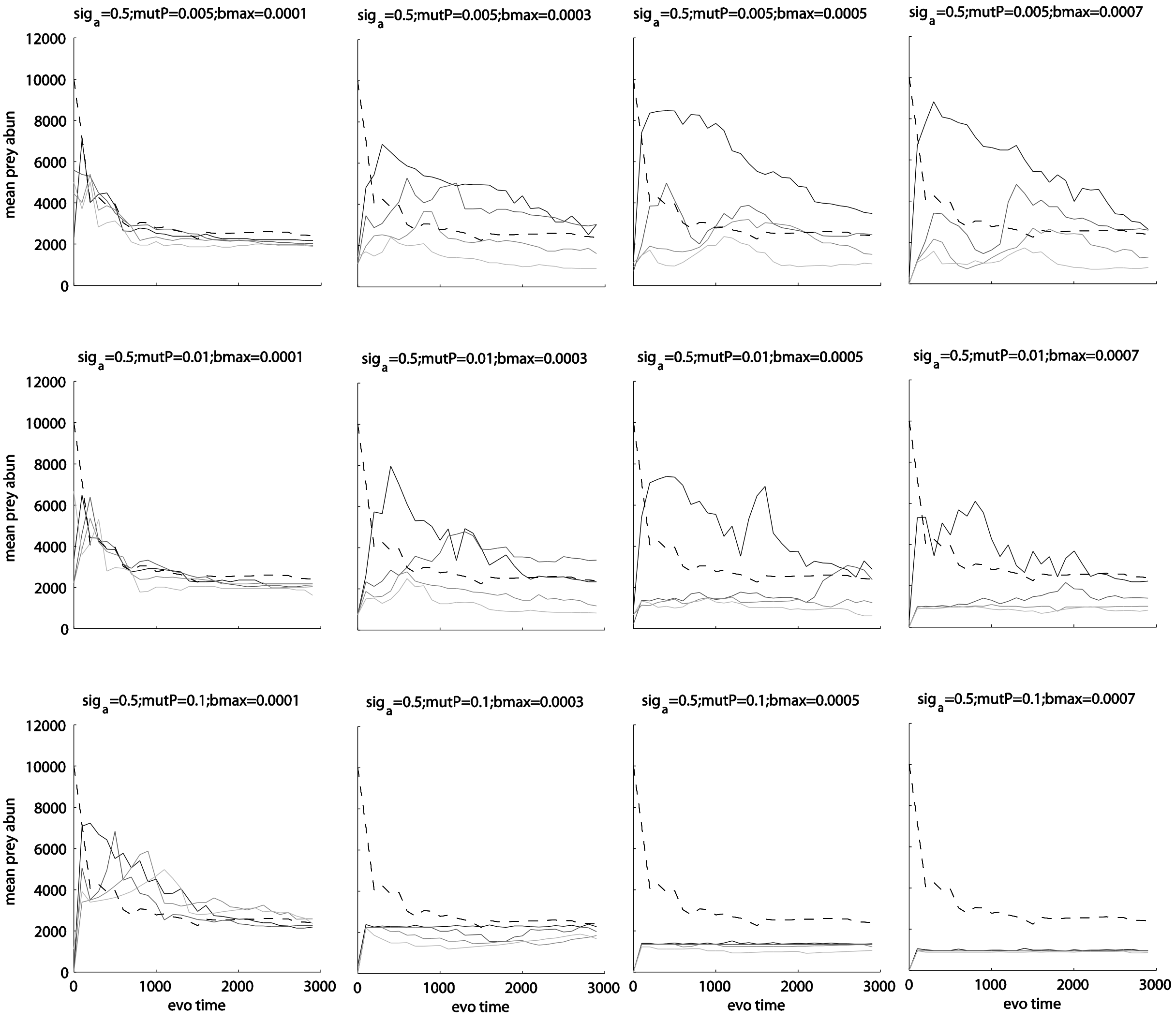

sig_a=0.5;mutP=0.005;bmax=0.0001
sig_a=0.5;mutP=0.005;bmax=0.0003
sig_a=0.5;mutP=0.005;bmax=0.0005
sig_a=0.5;mutP=0.005;bmax=0.0007
sig_a=0.5;mutP=0.01;bmax=0.0001
sig_a=0.5;mutP=0.01;bmax=0.0003
sig_a=0.5;mutP=0.01;bmax=0.0005
sig_a=0.5;mutP=0.01;bmax=0.0007
sig_a=0.5;mutP=0.1;bmax=0.0001
sig_a=0.5;mutP=0.1;bmax=0.0003
sig_a=0.5;mutP=0.1;bmax=0.0005
sig_a=0.5;mutP=0.1;bmax=0.0007
mean prey abun
0
2000
4000
6000
8000
10000
12000
evo time
0
1000
2000
3000

Figure S12

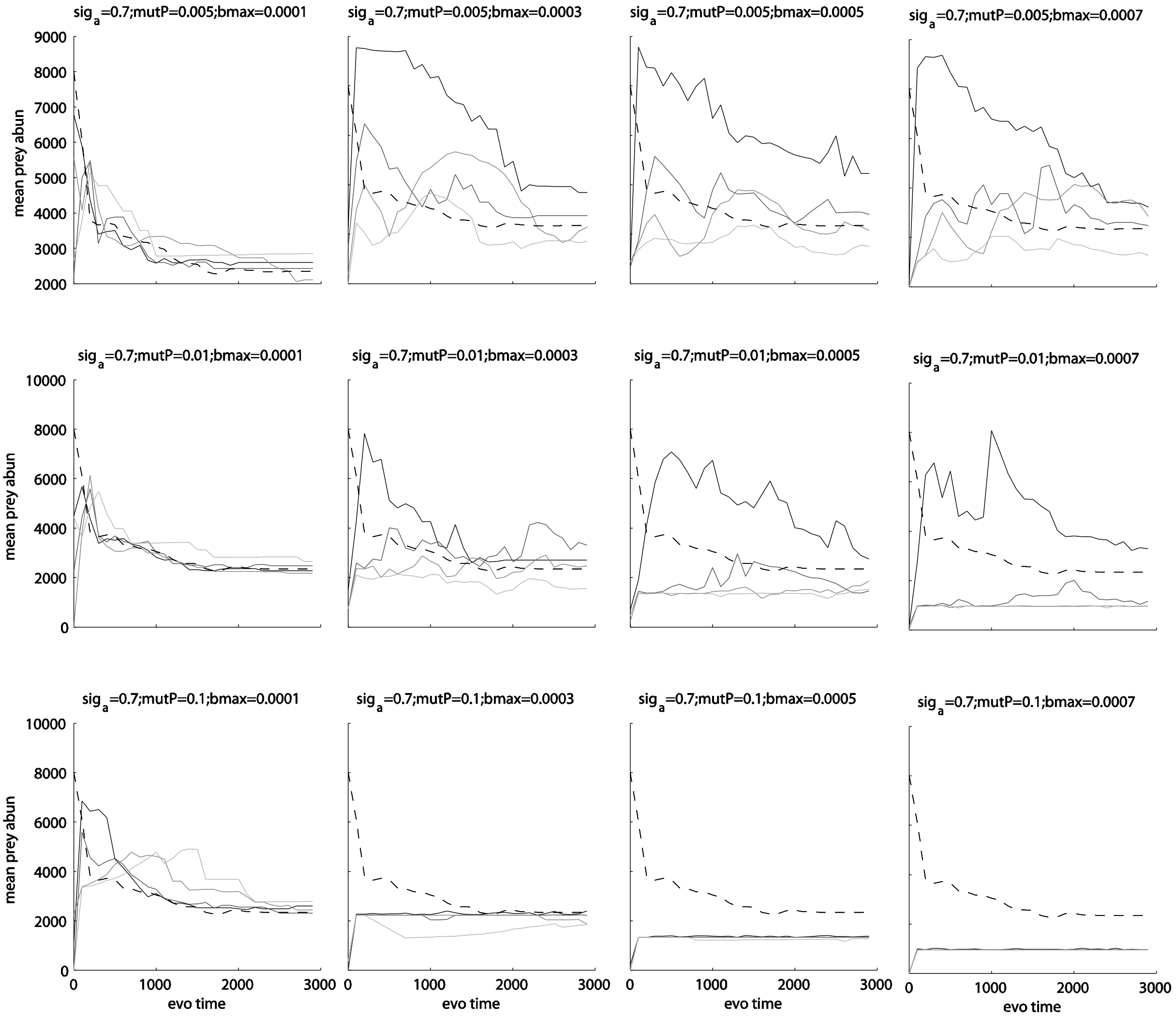
sig_a=0.7;mutP=0.005;bmax=0.0001
sig_a=0.7;mutP=0.005;bmax=0.0003
sig_a=0.7;mutP=0.005;bmax=0.0005
sig_a=0.7;mutP=0.005;bmax=0.0007
sig_a=0.7;mutP=0.01;bmax=0.0001
sig_a=0.7;mutP=0.01;bmax=0.0003
sig_a=0.7;mutP=0.01;bmax=0.0005
sig_a=0.7;mutP=0.01;bmax=0.0007
sig_a=0.7;mutP=0.1;bmax=0.0001
sig_a=0.7;mutP=0.1;bmax=0.0003
sig_a=0.7;mutP=0.1;bmax=0.0005
sig_a=0.7;mutP=0.1;bmax=0.0007
mean prey abun
evo time
0
1000
2000
3000
2000
3000
4000
5000
6000
7000
8000
9000
10000

Figure S13

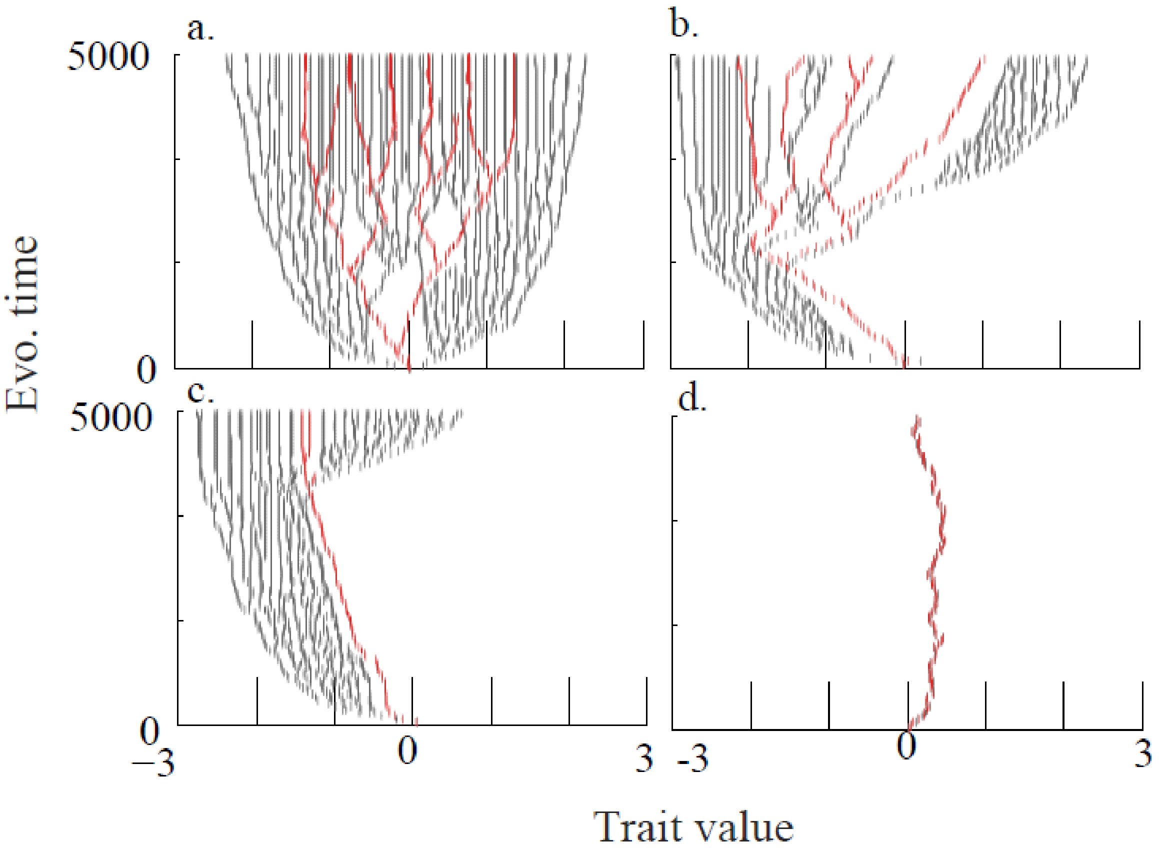

Figure S14

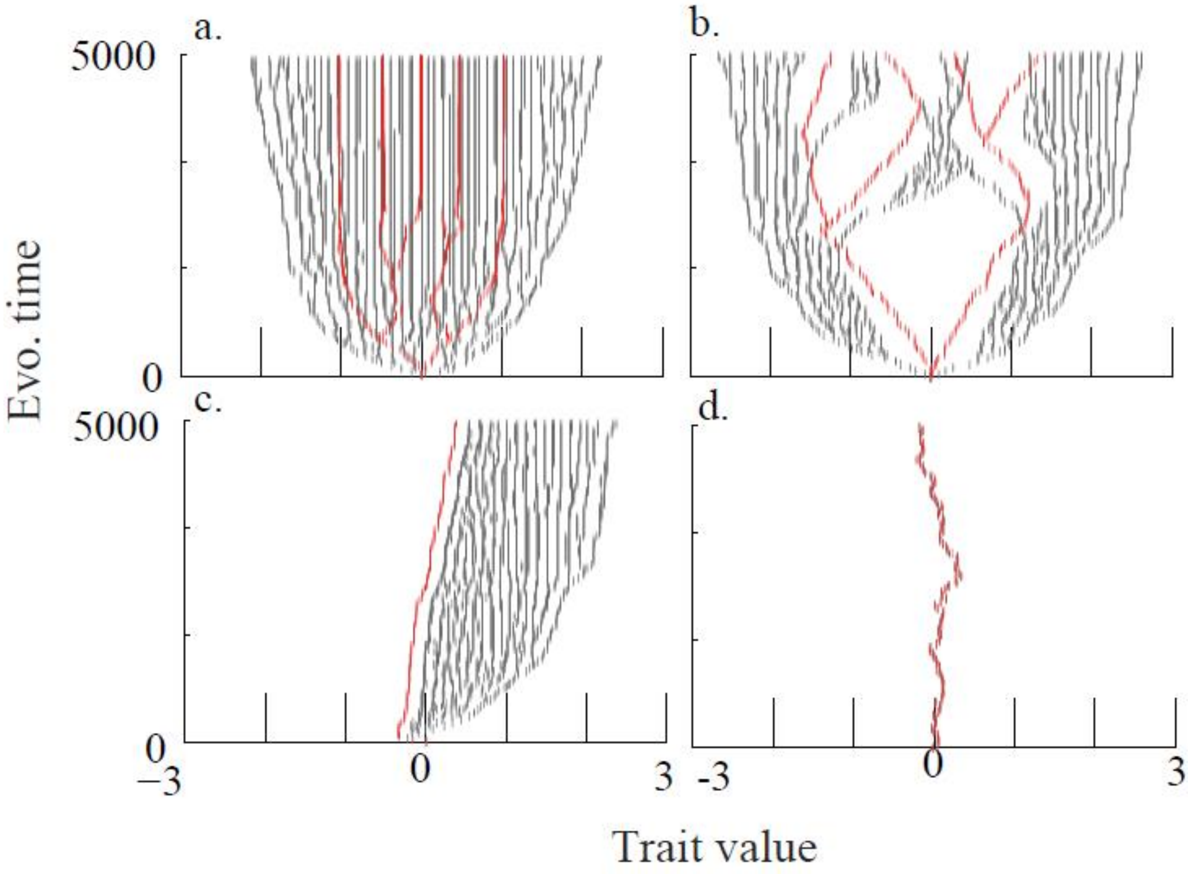

121 Figure S15

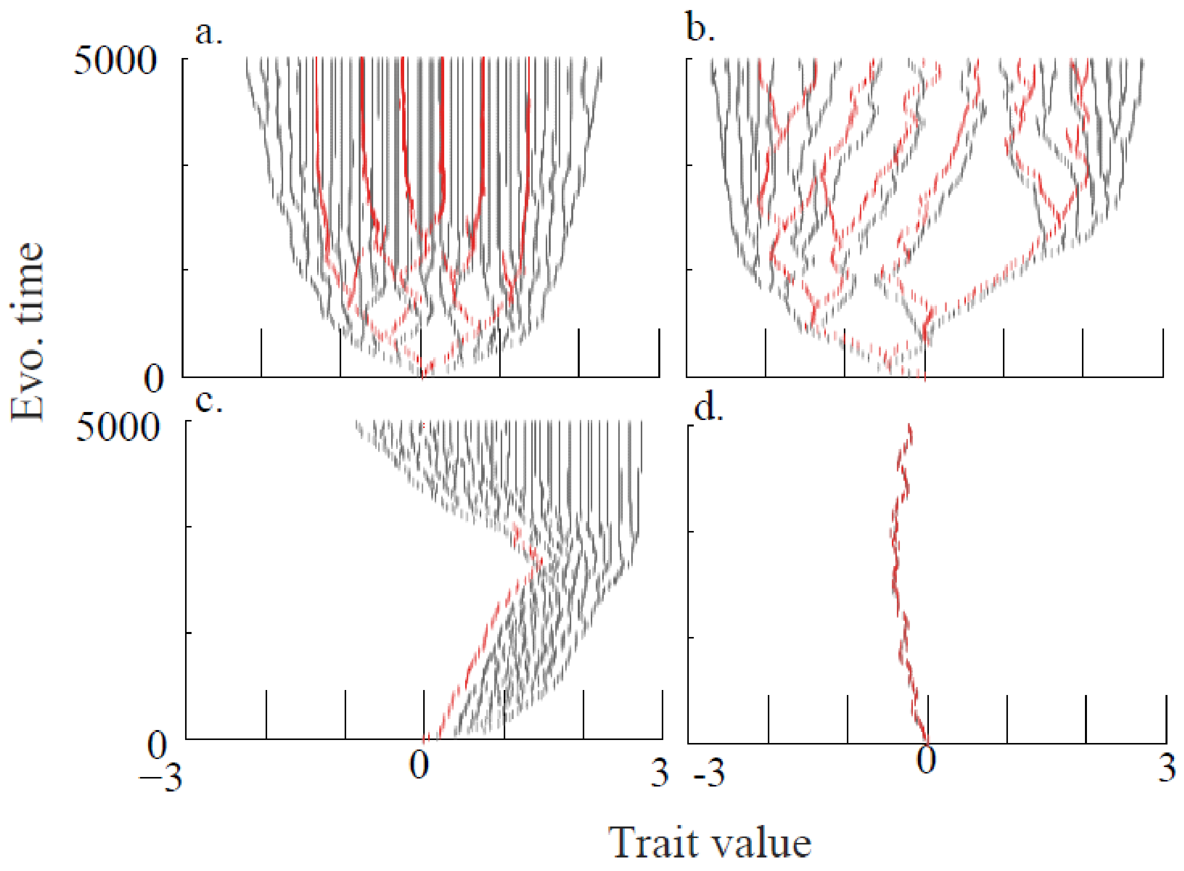

122
